# Child maltreatment as a transdiagnostic risk factor for the externalizing dimension: a Mendelian randomization study

**DOI:** 10.1038/s41380-024-02700-8

**Published:** 2024-08-22

**Authors:** Julian Konzok, Mathias Gorski, Thomas W. Winkler, Sebastian E. Baumeister, Varun Warrier, Michael F. Leitzmann, Hansjörg Baurecht

**Affiliations:** 1 Department of Epidemiology and Preventive Medicine, University of Regensburg, Regensburg, Germany; 2 Department of Genetic Epidemiology, University of Regensburg, Regensburg, Germany; 3 Institute of Health Services Research in Dentistry, University of Münster, Münster, Germany; 4 Department of Psychiatry, University of Cambridge, Cambridge, UK

**Keywords:** Psychology, Genetics, Psychiatric disorders

## Abstract

Observational studies suggest that child maltreatment increases the risk of externalizing spectrum disorders such as attention deficit hyperactivity disorder (ADHD), conduct disorder (CD), antisocial personality disorder (ASPD), and substance use disorder (SUD). Yet, only few of such associations have been investigated by approaches that provide strong evidence for causation, such as Mendelian Randomization (MR). Establishing causal inference is essential given the growing recognition of gene-environment correlations, which can confound observational research in the context of childhood maltreatment. Evaluating causality between child maltreatment and the externalizing phenotypes, we used genome-wide association study (GWAS) summary data for child maltreatment (143,473 participants), ADHD (20,183 cases; 35,191 controls), CD (451 cases; 256,859 controls), ASPD (381 cases; 252,877 controls), alcohol use disorder (AUD; 13,422 cases; 244,533 controls), opioid use disorder (OUD; 775 cases; 255,921 controls), and cannabinoid use disorder (CUD; 14,080 cases; 343,726 controls). We also generated a latent variable ‘common externalizing factor’ (EXT) using genomic structural equation modeling. Genetically predicted childhood maltreatment was consistently associated with ADHD (odds ratio [*OR*], 10.09; 95%-CI, 4.76–21.40; *P* = 1.63 × 10^−09^), AUD (*OR*, 3.72; 95%-CI, 1.85–7.52; *P* = 2.42 × 10^−04^), and the EXT (*OR*, 2.64; 95%-CI, 1.52–4.60; *P* = 5.80 × 10^−04^) across the different analyses and pleiotropy-robust methods. A subsequent GWAS on childhood maltreatment and the externalizing dimension from Externalizing Consortium (EXT-CON) confirmed these results. Two of the top five genes with the strongest associations in EXT GWAS, CADM2 and SEMA6D, are also ranked among the top 10 in the EXT-CON. The present results confirm the existence of a common externalizing factor and an increasing vulnerability caused by child maltreatment, with crucial implications for prevention. However, the partly diverging results also indicate that specific influences impact individual phenotypes separately.

## Introduction

Several lines of research indicate high comorbidity among externalizing psychopathologies and significant heritability of a common externalizing factor [1, 2]. This common externalizing factor encompasses disinhibition, impulsivity, antisocial-aggressive behavior as well as substance (ab)use [1]. Clinically, the externalizing spectrum comprises attention-deficit/hyperactivity disorder (ADHD), conduct disorder (CD), antisocial personality disorder (ASPD), and substance use disorders (SUD) [3]. Multiple studies have demonstrated a shared genetic basis for these disorders [4, 5].

**Fig. 1:**
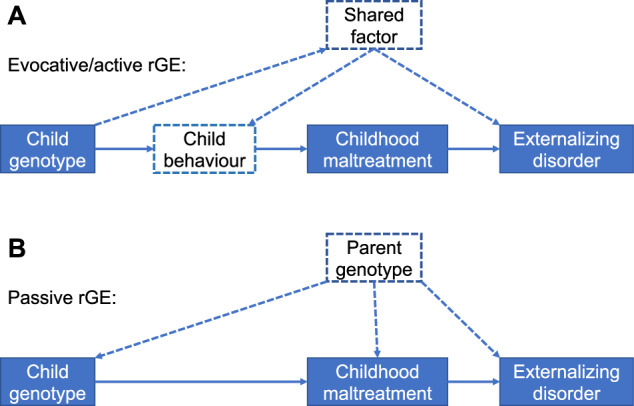
Three types of gene-environment correlations (rGE) potentially rising correlated pleiotropy in causal modeling. In evocative/active rGE (**A**), connection between child genotypes and exposure is conditioned on child behavior potentially resulting in a pathway to the outcome independent of the exposure. In passive rGE (**B**), causal estimation could be confounded by parent genotype. Dashed arrows symbolize potential confounding or pleiotropic pathways, solid arrows represent causal pathways.

One of the main candidates influencing the etiology of psychiatric disorders is childhood maltreatment. Childhood maltreatment encompasses emotional, sexual, physical abuse, and emotional and physical neglect [6]. A wide range of observational studies, including case-control designs, showed that childhood maltreatment increases risks for ADHD [7], ASPD [8] and SUD [9]. Twin and family studies demonstrated that childhood maltreatment has a heritability of 6 to 62% [10, 11], depending on the subtype. These findings appear surprising, given that childhood maltreatment is an environmental, and thus potentially modifiable, determinant. However, investigations have shown that such heritability is derived from gene-environment correlations (rGE) with passive (i.e., family environment influenced by shared genetic factors of parents and infants), evocative/ reactive (i.e., parental style partly caused by hereditary characteristics of the offspring), and active (i.e., selection of specific contexts influenced by heritable traits) subtypes [12] (see Fig. 1). This raises the question of whether the relationship between childhood maltreatment and externalizing disorders is causal or is mainly driven by rGE. In the second case, risk for psychiatric diseases will only slightly be modified by childhood maltreatment, with crucial implications for prevention and treatment. Besides experimental and quasi-experimental designs controlling for genetic confounding, Mendelian Randomization (MR) [13] can be used to assess causal effects of environmental risk factors on mental health outcomes under certain key assumptions, even in the presence of rGE [14]. However, in the presence of rGE, particular attention should be paid to methods (e.g., Causal analysis using summary effect estimates, CAUSE) [15] reducing bias (e.g., correlated pleiotropy) arising from genetic correlation [14, 16]. Correlated pleiotropy occurs when genetic variants influence both exposure and outcome through a heritable shared factor (see Fig. 1), which can bias MR analysis.

To the best of our knowledge, only one two-sample MR study investigated the effect of childhood maltreatment on ADHD indicating an increasing risk [12]. Evidence from MR studies for other externalizing disorders (e.g., ASPD, SUD) is sparse. Furthermore, observational studies suggest an effect of externalizing problems on the risk of childhood maltreatment [7], which so far has only been investigated in ADHD patients using an MR approach. Additionally, no investigation has examined the externalizing factor reflecting common variation across externalizing disorders and a shared genetic basis. Evidence for childhood maltreatment as a transdiagnostic risk factor would have crucial implications for prevention strategies and programs (e.g., target individuals).

The current study aimed at investigating the causal relationship between childhood maltreatment and the risk of externalizing disorders accounting for rGE. In addition, we were interested in examining whether such a relationship also exists for a common externalizing factor reflecting comorbidity and continuity of externalizing disorders over the lifespan.

## Materials and methods

Single-nucleotide polymorphisms (SNPs) were used as instrumental variables (IVs) to estimate the effect of the exposure on the outcomes unbiased from any unobserved confounding under the condition of valid IVs. IVs are valid if the following three assumptions are fulfilled. (i) The genetic variant is associated with the exposure (relevance assumption), (ii) the variant-outcome association is independent of a potential confounder (exchangeability assumption) and (iii) the genetic variant influences the outcome exclusively through the exposure, independent of any horizontal pleiotropy i.e., independent of any confounder or direct effect (exclusion restriction). Furthermore, several complementary analyses were conducted to evaluate potential biasing effects of correlated and uncorrelated horizontal pleiotropy as well as reverse causation [13, 17].

### Selection of instrumental variables for childhood maltreatment

Linkage-disequilibrium-(LD)-independent SNPs associated with childhood maltreatment were selected from a GWAS in 143,473 participants [12] (exclusively from UK Biobank to avoid overlap with the outcome GWAS) (Supplementary Table S1) at a genome-wide level of significance (*P* value < 5 × 10^−8^). Within this clumping algorithm, we excluded SNPs that exhibited strand ambiguity and had a minor allele frequency of less than 0.01. We applied a threshold of r^2^ at 0.001 and employed a window size of 10 Mb. Then, we calculated the *F*-statistic and the proportion of the variance explained by childhood maltreatment by summarizing values from all SNPs. In the UK Biobank, participants completed the five-item Childhood Trauma Screener [18], which is a retrospective assessment. This screener includes one question for each of the five trauma subtypes (emotional, sexual, and physical abuse, and emotional and physical neglect), with responses ranging from 0 (never true) to 4 (very often true), resulting in total scores ranging from 0 to 20. The continuously coded total score was included in the GWAS for childhood maltreatment. In the original study, the authors identified 16 significant SNPs associated with 9 genomic risk loci [12] for the UK Biobank only data set.

### Genome-wide association study summary statistics for the six externalizing disorders

To maintain consistency in the used phenotype definitions, we focused on GWAS for externalizing disorders clinically diagnosed by ICD or DSM, resulting in partly diminished numbers of cases due to limited data availability. For ADHD, we used data from a meta-analysis of samples of the Psychiatric Genomics Consortium (PGC) and the iPSYCH project totaling 20,183 cases and 35,191 controls [19]. For CD and ASPD, GWAS summary statistics stemmed from the FinnGen Consortium with 451 cases and 256,859 controls and 381 cases and 252,877 controls, respectively [20]. The GWAS on AUD and OUD were also conducted by the FinnGen Consortium with 13,422 cases and 244,533 controls and 775 cases and 255,921 controls, respectively [20]. Summary data statistics for cannabinoid use disorder (CUD) were derived from the PGC Substance Use Disorders working group, iPSYCH, and deCODE, with 14,080 cases and 343,726 controls [21] (Supplementary Table S1, S2). Manhattan and quantil-quantil (Q-Q) plots of the used GWAS summary data are depicted in Supplementary Fig. S3, S4.

### Statistical analyses

#### Shared genetic basis of the externalizing phenotypes: the externalizing factor (EXT)

Using GenomicSEM, a common factor model and a commonfactorGWAS function were performed with a diagonally weighted least squares (DWLS) estimation, integrating the GWAS of the six externalizing phenotypes to a common factor GWAS. We assessed the model fit using the comparative fit index (CFI), standardized root mean square residual (SRMR), and the standardized loading of the common externalizing factor on the specific phenotypes. CFI scores of ≥0.90 indicate adequate fit, while values of ≥0.95 imply a good model fit [22]. SRMR values below 0.10 suggest an adequate model fit, values less than 0.05 point to a good fit [23]. SNPs with a significant heterogeneity test (*P* < 0.05) were excluded from the common factor GWAS, and the effective sample size estimation was conducted with a minor allele frequency between 0.4 and 0.1. In the following MR analyses, this more holistic phenotype was termed ‘externalizing factor’ (EXT). To determine the individual importance of each externalizing disorder in shaping the overall EXT, we stepwise excluded each externalizing disorder and correlated these models in a leave-one-out-analysis. Additionally, heterogeneity analysis was applied using *Q*_SNP_ statistic to test whether each SNP-externalizing-disorder association is conditioned on the common EXT. A significant Q_SNP_ heterogeneity statistic indicated a pathway from the genetic variant to the externalizing disorder, independent of the common EXT.

To identify independent significant SNPs and corresponding genomic risk loci associated with the EXT, we used Functional Mapping and Annotation (FUMA) [24]. Within Multi-marker Analysis of GenoMic Annotation (MAGMA) gene-based association analysis, genome-wide significant SNPs were initially mapped to 19,176 protein-coding genes, and the SNPs within each gene were collectively tested for their association with EXT. Significance threshold for this analysis was Bonferroni corrected and defined at 2.61 × 10^−6^.

#### Primary analysis

Power analyses were performed following Brion et al. [25] (for detailed information see Supplementary Material 1). In MR analysis, Wald ratios (i.e. ratio of coefficients method) were calculated by dividing the logistic regression coefficient of the SNP-outcome associations by the regression coefficient of the SNP-exposure associations for each genetic variant selected from exposure GWAS. The delta method was used for standard error calculation. The ratio estimates (presumed to be linear on the log odds ratio scale) were subsequently combined using a multiplicative random effects model in an inverse variance weighted estimate (IVW) over all SNPs. The odds ratio is obtained by using the ratio estimates as exponent to the basis e [13, 17]. We used a false-discovery rate (FDR) corrected threshold of .05 (*q*-value) to account for multiple testing.

#### Sensitivity analysis: test for directional pleiotropy and pleiotropy-robust methods

For outlier diagnostics indicating invalid instruments (violation of the exclusion restriction assumption), the *Q* and *I²* statistic were used to test globally for heterogeneity. Additionally, leave-one out analysis was conducted to check whether the overall estimate was driven by a specific SNP. Furthermore, the MR Egger intercept test was conducted to evaluate potential influences of directional pleiotropic effects (i.e., the average pleiotropic effect deviates from zero and is shifted in one direction). Weighted median, radial regression MR, and MR pleiotropy residual sum and outlier (MR PRESSO) [26] were conducted as pleiotropy-robust methods.

#### Causal analysis using summary effect estimates (CAUSE) and test for reverse causation

Facing the low effective sample size in some summary statistics, we employed Causal Analysis Using Summary Effect estimates (CAUSE) that uses all genetic variants for causal estimation, thereby increasing statistical power [15]. CAUSE aims to distinguish between a causal effect of the exposure on the outcome (i.e., correlation of between all SNP-exposure and SNP-outcome estimates of all genetic variants associated with the exposure) from correlated pleiotropy induced by a shared (unknown) factor (i.e., correlation only in a subset of variants). Causal inference was obtained by a Bayesian approach comparing the two nested models, the causal model allowing a nonzero causal effect and the sharing model with causal effect fixed at zero [15].

Reverse causation analysis, i.e. exposure and outcome were swapped, was also carried out by CAUSE due to the low number of genetic instruments and effective sample size in some outcome GWAS.

#### Replication analysis

As replication analysis, we used SNPs from a second childhood maltreatment GWAS of 15,651 individuals of European descent from the Avon Longitudinal Study of Parents and Children (ALSPAC) [27], Adolescent Brain Cognitive Development Study (ABCD) [28], and Generation R [29] recording childhood maltreatment prospectively using multiple questionnaires at multiple instances (majority parent report, several self-report) [12]. Since this GWAS for childhood maltreatment is also of limited statistical power, we again employed the CAUSE approach which increases power by incorporating all genetic variants.

In addition, we rerun the primary analysis replacing the estimated EXT by the externalizing factor obtained from a GWAS conducted by the Externalizing Consortium (EXT-CON) excluding 23andme [5, 30] with 579 genome-wide significant SNPs. These SEM-GWAS employed a broader definition of externalizing traits for inclusion and did not limit their analysis solely to ICD-coded disorders.

All analyses were performed using the packages MRInstruments (0.3.2), MendelianRandomization (0.6.0), TwoSampleMR (0.5.6), MRPRESSO (1.0) and cause (1.2.0) in R, version 4.2.2 (2022/10/31). We report the methods and results following the STROBE-MR (Strengthening the Reporting of Observational studies in Epidemiology – Mendelian randomization) statement [31].

## Results

### Shared genetic basis of the externalizing phenotypes: the externalizing factor (EXT)

**Fig. 2:**
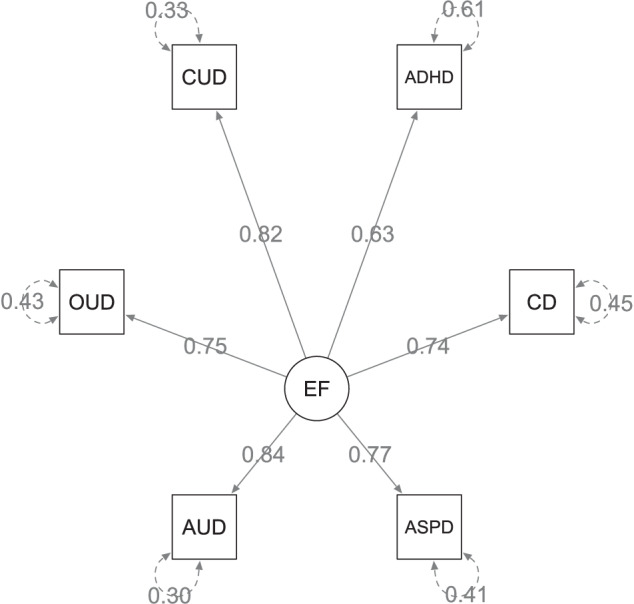
Path diagram for the externalizing factor (EF) with standardized loadings of attention deficit hyperactivity disorder (ADHD), conduct disorder (CD), antisocial personality disorder (ASPD), alcohol use disorder (AUD), opioid use disorder (OUD), and cannabinoid use disorder (CUD). The rectangles symbolize the indicators, the latent common factor is presented as circle. Single headed arrows indicate the direction of the regression effect with the standardized loadings. Double headed arrows reflect standardized residuals.

**Fig. 3:**
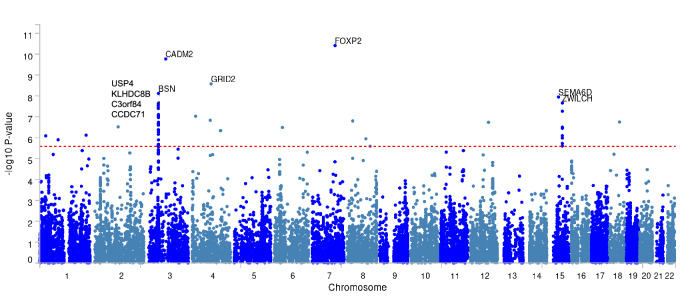
Manhattan plot of the genes analyzed for association in the MAGMA gene-based association analysis. The 10 significant genes with the strongest association are labeled. The red dashed line indicates Bonferroni corrected significance threshold at 2.61 × 10^−6^.

The common factor model exhibited a CFI of 1 and a SRMR of 0.097, indicating a good model fit (CFI > 0.95, SRMR < 0.10). All indicators showed standardized loadings on the EXT over 0.60, with strong loadings for AUD (0.84, *SE* = 0.05, *p* = 2.31 × 10^−54^) and CUD (0.82, *SE* = 0.06, p = 2.57 × 10^−40^), moderate loadings for ADHD (0.63, *SE* = 0.05, *p* = 1.29 × 10^−31^), CD (0.74, *SE* = 0.06, *p* = 2.37 × 10^−13^), ASPD (0.77, *SE* = 0.09, *p* = 2.31 × 10^−54^), and OUD (0.75, *SE* = 0.11, *p* = 1.15 × 10^−12^) (see Fig. 2). The Supplementary Fig. S1 depicts the genetic correlation matrix of the indicator GWAS. The EXT explained 39.2% of the variance of ADHD, 54.6% of CD, 58.6% of ASPD, 69.7% of AUD, 57.0% of OUD, and 66.9% of CUD. The chi-squared-test yielded a non-significant result (χ^2^(*df* = 9) = 6.91, *P* = 0.65), indicating a better fit for the common model to the observed GWAS data. This also confirmed the existence of a shared genetic basis of the six externalizing phenotypes. Q_SNP_ analysis identified four SNPs displaying remarkable heterogeneity (*P* < 5 × 10^−08^), but only one overlapped with the genome-wide significant variants associated with the EXT, indicating no pleiotropic effect among individual externalizing disorders, independent of the common EXT. After excluding SNPs with a significant heterogeneity test (*P* < 0.05), the common GWAS comprised 6,004,696 SNPs associated with the EXT. Each individual externalizing disorders notably contributed to the EXT, as evidenced by comparable correlation between the different leave-one-out models (*r*_*g*_*:* 0.90–0.99, *SE:* 0.01–0.17). The common factor GWAS exhibited 45 independent genome-wide significant genetic variants with 39 genomic risk loci. Figure 3 illustrates the top 10 genes with the strongest associations (see also Supplementary Table S3). The top five genes include forkhead box P2 (FOXP2), cell adhesion molecule 2 (CADM2), glutamate ionotropic receptor delta type subunit 2 (GRID2), bassoon presynaptic cytomatrix protein (BSN), and semaphoring 6D (SEMA6D). These genes were previously associated among others with externalizing disorders and other psychiatric [32, 33] as well as addiction related traits [34, 35].

### Power analysis

The analysis had a power of ≥90% to detect a minimum OR of 2.00 for ADHD, 5.00 for CD, >5.00 for ASPD, 1.80 for AUD, 4.00 for OUD, and 1.80 for CUD (Supplementary Table S4).

### Primary analysis

**Fig. 4:**
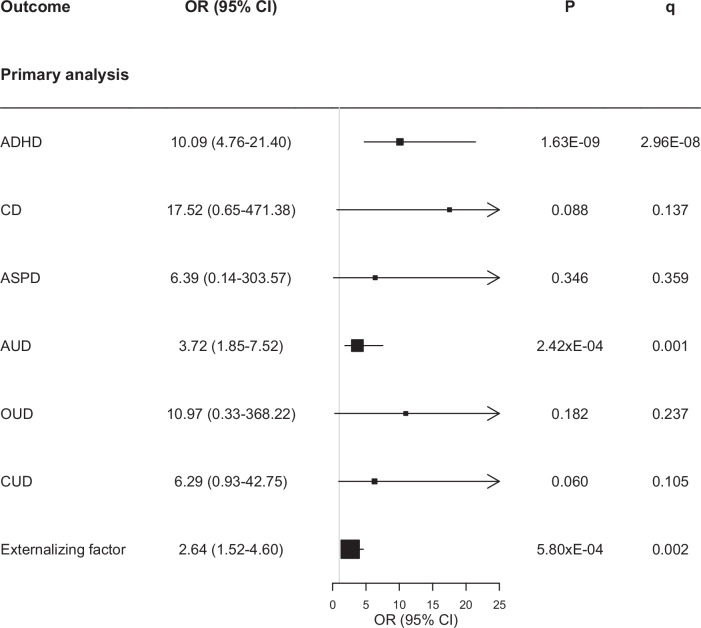
Mendelian Randomization estimates for association between genetically instrumented childhood maltreatment and attention deficit hyperactivity disorder (ADHD), conduct disorder (CD), antisocial personality disorder (ASPD), alcohol use disorder (AUD), opioid use disorder (OUD) and cannabinoid use disorder (CUD). CI confidence interval, OR odds ratio, *P* = *p* value, q = adjusted *p* values using a FDR approach.

The 6 selected genetic instruments explained 0.2% of the variability of the exposure, with a minimum *F*-statistic of 29.85 (Supplementary Table S5). The Standard IVW MR analysis showed significant effects corrected for multiple testing of childhood maltreatment on ADHD, AUD and the EXT. The effects of childhood maltreatment on CD, ASPD, OUD and CUD did not reach statistical significance (see Fig. 4 and Supplementary Table S6).

### Sensitivity analysis: test for directional pleiotropy and pleiotropy-robust methods

For CUD and the EXT, we observed heterogeneity between the Wald ratios of the IVW estimates suggesting pleiotropy. However, the MR Egger intercept test indicated no directional pleiotropy for both outcomes (Supplementary Table S7). Visual inspection of funnel plots (Supplementary Fig. S2) supported these findings and showed no strong deviation from symmetrical distributions, indicating balanced rather than directional pleiotropy and does not distort causal estimation. The applied random effects IVW model accounts for additional heterogeneity. Consistent with this, pleiotropy-robust methods (weighted median, radial regression MR, and MR PRESSO) showed similar results to the random effects IVW for all phenotypes (Supplementary Table S6). Additionally, the stepwise leave-one-out analysis did not reveal any genetic variant as a leverage point with high influence (see Supplementary Table S8).

### Causal analysis using summary effect estimates (CAUSE) and test for reverse causation

The CAUSE approach confirmed the significant causal associations of childhood maltreatment with ADHD (*OR* = 1.90, 95% credible interval (*CredIn*): 1.23–2.91, *P* = 0.004), ASPD (*OR* = 9.12, 95% *CredIn*: 1.07–79.04; *P* = 0.045), and the EXT (*OR* = 1.34, 95% *CredIn*: 1.14–1.67; *P* = 1.43 × 10^−07^). However, there was no significant difference between the causal and shared model for CD (*OR* = 3.94, 95% *CredIn*: 0.56–25.79; *P* = 0.153), AUD (*OR* = 1.35, 95% *CredIn*: 0.84–2.14; *P* = 0.201), OUD (*OR* = 2.41, 95% *CredIn*: 0.51–11.14; *P* = 0.260), and CUD (*OR* = 2.36, 95% *CredIn*: 0.87–1.42; *P* = 0.179) suggesting no causal association of child maltreatment on those traits.

Reverse causation analyses suggested only a significant causal influence of ADHD (*OR* = 1.01, 95% *CredIn*: 1.00–1.02, *P* = 8.85 × 10^−05^) on childhood maltreatment with estimates close to one, but not for CD (*OR* = 1.01, 95% *CredIn*: 0.99–1.04, *p* = 0.514), ASPD (*OR* = 1.01, 95% *CredIn*: 0.99–1.03, *P* = 0.327), AUD (*OR* = 1.02, 95% *CredIn:* 0.99–1.04, *P* = 0.050), OUD (*OR* = 1.00, 95% *CredIn:* 0.98–1.02, *P* = 1.00), CUD (*OR* = 1.00, 95% *CredIn:* 0.98–1.02, *P* = 1.00), and the EXT (*OR* = 1.01, 95% *CredIn:* 0.99–1.04, *P* = 0.514).

### Replication analysis

Analysis using an independent childhood maltreatment GWAS replicates only the finding of a causal effect of childhood maltreatment on the EXT (*OR* = 1.31, 95% *CredIn*: 1.04–1.64, *P* = 0.021), but not on ADHD and AUD. The null association with all other externalizing disorders was confirmed (Supplementary Table S9).

In a second replication we replaced the summary statistics of the calculated EXT by the EXT-CON [5, 30]. Again, genetically predicted childhood maltreatment is causally associated with the EXT-CON (1.69, 95%*CI*: 1.30–2.21, *p* = 8.83 × 10^−05^).

## Discussion

The current study investigated the causal association between childhood maltreatment and the risk for externalizing disorders such as ADHD, CD, ASPD, and substance use disorders such as AUD, OUD and CUD. Genetically predicted childhood maltreatment strongly increased the risk for ADHD, and AUD in later life, aligning with previous observational studies of ADHD [7] and alcohol use disorder patients [36]. In contrast to observational methods, MR methods have the advantage of effectively accounting for effects of unobserved confounding factors. This point is important to emphasize, as there are other potential (confounding) factors (e.g., socioeconomic status, rGE) that contribute to both childhood maltreatment and mental disorders. Furthermore, the causal effect of childhood maltreatment on externalizing disorders is supported by animal studies that infer causality through experiments that are ethically unacceptable in humans. These studies demonstrated that early childhood stress influences alcohol and drug consumption and other behavioral differences in monkeys and rodents [37, 38].

We found no causal effect of childhood maltreatment on the risk for development of CD, ASPD, OUD, and CUD. It is important to note that GWAS for CD, ASPD and OUD exhibited a rather small proportion of cases compared to controls, which led to limited power to detect differences. Thus, we performed the CAUSE approach using all genetic variants for causal estimation, thereby increasing statistical power. Using CAUSE, childhood maltreatment is besides ADHD and AUD also causally related with ASPD. However, this causal relation was not identified by IVW analysis, which may be due to low power. Further research using GWAS with a larger effective sample size is needed for clarification.

The main methodological challenge in the presence of rGE is pleiotropy, which can lead to an inaccurate causal estimation. MR PRESSO and MR egger rely on the InSIDE assumption (i.e., pleiotropic effect has to be independent of the instrument strength). Both methods can deal with horizontal pleiotropy (i.e., independent genetic effects on exposure and outcome), but not correlated pleiotropy (i.e., variants influence exposure and outcome through shared genetic factor) induced by rGE [26, 39]. Median based estimations are robust to all forms of pleiotropy, albeit to a lesser extent. In contrast, the CAUSE approach distinguishes the causal effect of uncorrelated and correlated pleiotropy induced by rGE [15]. Across the different pleiotropy-robust methods, including CAUSE, childhood maltreatment was consistently associated with risk for the EXT, confirming a causal effect despite rGE. While CAUSE models a single unobserved factor to account for shared and correlated effects, other approaches directly incorporate family and sibling data to control for biasing family effects [40]. Since we did not have access to parental genotypic data, we were unable to perform within-family MR. Future studies should aim to replicate our findings using within-family designs to further validate the results.

Our analyses also indicated reverse causation between childhood maltreatment and ADHD. This is consistent with a previous MR using partially overlapping data sources [12]. Not surprisingly, externalizing behavior and temperament are associated with inadequate parental response, which, along with certain other factors (e.g., low parental self control, socioeconomic status), may promote maladaptive parent-child interactions and childhood maltreatment. To date, only a few observational and MR studies have shown this finding [7, 12].

Our study demonstrated for the first time that childhood maltreatment leads to a significant susceptibility to the common EXT. This result was also confirmed with the common factor GWAS from the Externalizing Consortium (EXT-CON) [5, 30]. Of note, two of the top five genes associated with EXT in our study (CADM2, SEMA6D) also ranked among the top 10 in the EXT-CON GWAS. In contrast to the EXT-CON model, we incorporated also antisocial traits into our EXT model, a well-established facet of the externalizing dimension in various research lines [1, 2]. MR analyses of both independent datasets revealed a robust causal relationship, however with divergent odds ratios, possibly attributed to the limited number of genetic instruments due to small sample size. Previous research supports the notion of a highly heritable externalizing factor (*h*^*2*^: 81–84%) [1, 2] underlying externalizing phenotypes. Our structural equation modeling revealed a substantial unexplained variance in specific phenotypes, indicating additional factors (i.e., what distinguishes ADHD from ASPD). This is in line with the hierarchical model of the externalizing spectrum [1, 2], positing the existence of both general and specific etiological factors. Furthermore, the divergent effect estimates for different disorders in our study also suggest the existence of additional specific factors contributing to the manifestation of individual disorders.

Moreover, when regarding childhood maltreatment as a comprehensive risk factor, it raises the possibility that it might also play a role in increasing susceptibility within the internalizing dimension, such as depression and anxiety. We plan to investigate this aspect in an upcoming study, where we will assess the impact on both the externalizing and internalizing dimensions.

Our study has several limitations. Firstly, the genetic variants we selected explained only a small fraction of the overall variance in childhood maltreatment. Consequently, the GWAS for childhood maltreatment revealed a relatively low number of genome-wide significant SNPs as instruments for MR analyses. Nonetheless, our chosen instruments demonstrated a minimum F-statistic of 29.85, which indicates no evidence of weak instrument bias, reinforcing the reliability of our selected instruments. Furthermore, the complementary CAUSE approach utilizes all available genetic variants, enhancing statistical power. Secondly, as previously mentioned, the statistical power of specific analyses, particularly those related to CD, ASPD, and OUD, was constrained by the relatively small number of cases falling below 1000. However, the incorporation of the complementary CAUSE approach allowed us to generate better powered causal estimates, thereby reducing the false-positive rate. Thirdly, despite exclusively including GWAS on ICD-coded outcomes, it’s possible that variation in measurement methods existed across the different cohorts.

## Conclusion

The current study demonstrated that childhood maltreatment ranks among the etiological influences of the common externalizing factor, besides the existence of factors contributing to the specific phenotypes separately. This has crucial implications for prevention strategies. First, it underlines the importance of primary and secondary prevention services, as childhood maltreatment has now been established as a vulnerability factor for numerous psychiatric conditions. Second, our findings support the use of a comprehensive understanding of externalizing disorders in the development of tertiary prevention services for childhood maltreatment, regardless of the onset of externalizing disorders. For instance, interventions could focus on negative emotionality, low fearfulness and effortful control [41] or target the biological changes (e.g., altered cortisol reactivity), also associated with early stages of the externalizing spectrum [42].

## Supplementary information

Supplements: Child maltreatment as a transdiagnostic risk factor for the externalizing dimension: A Mendelian Randomization study

## Data Availability

GWAS summary statistics for attention deficit hyperactivity disorder and cannabinoid use disorder from the Psychiatric Genomics Consortium are available at https://pgc.unc.edu/for-researchers/download-results/, for conduct disorder, antisocial personality disorder, alcohol use disorder, opioid use disorder from the FinnGen Consortium at https://www.finngen.fi/en/access_results/. The R code is available from the corresponding author on request.
