# Supplementary material for: Child maltreatment as a transdiagnostic risk factor for the externalizing dimension: a Mendelian randomization study
Source: Mol Psychiatry. 2024 Aug 22;30(2):567–73. doi: 10.1038/s41380-024-02700-8 (PMC11746131; doi:10.1038/s41380-024-02700-8)
Supplement: Supplementary file 1 — Supplements: Child maltreatment as a transdiagnostic risk factor for the externalizing dimension: A Mendelian Randomization study [file 41380_2024_2700_MOESM1_ESM.docx]

**Supplements: Child maltreatment as a transdiagnostic risk factor for the externalizing dimension: A Mendelian Randomization study**

Julian Konzok, PhD^1^*, Mathias Gorski, PhD^2^, Thomas W. Winkler, PhD^2^, Sebastian E. Baumeister, PhD^3^, Varun Warrier, PhD^4^, Michael F. Leitzmann, PhD, MD^1^, Hansjörg Baurecht, PhD^1^

^1^ Department of Epidemiology and Preventive Medicine, University of Regensburg, Germany

^2^ Department of Genetic Epidemiology, University of Regensburg, Regensburg, Germany

^3^ Institute of Health Services Research in Dentistry, University of Münster, Münster, Germany

^4^ Department of Psychiatry, University of Cambridge, Cambridge, UK

*Corresponding author: [julian.konzok@ukr.de](mailto:julian.konzok@ukr.de) (Julian Konzok)

# Supplementary Material 1

*Power analysis following Brion et al.:* This method aligns with asymptotic theory and derives power estimates using the non-centrality parameter from the corresponding asymptotic χ2-distribution. Additional parameters considered in the power calculations encompass the proportion of explained variance in the exposure attributable to the selected instrumental variables (IVs), the sample size of the outcome GWAS and its proportion of cases.

# Table S1 Overview of the used datasets

Note. ADHD = Attention deficit hyperactivity disorder, CD = Conduct disorder, ASPD = antisocial personality disorder, AUD = Alcohol use disorder, OUD = Opioid use disorder, CUD = Cannabinoid use disorder.

| Study | Phenotype | N cases | N controls | Data available |
| --- | --- | --- | --- | --- |
| Exposure | | | | |
| Meta-analysis of childhood maltreatment using data from the UK Biobank (1) | Childhood maltreatment | 143,473 | | data available |
| Replication dataset | | | | |
| GWAS meta-analysis from Avon Longitudinal Study of Parents and Children (ALSPAC), Adolescent Brain Cognitive Development Study (ABCS), and Generation R (1) | Childhood maltreatment | 15 651 | | data available |
| Outcome | | | | |
| PGC, iPSYCH project (2) | ADHD | 20,183 | 35,191 | data available |
| FinnGen Consortium (3) | CD | 451 | 256,859 | data available |
| FinnGen Consortium (3) | ASPD | 381 | 252,877 | data available |
| FinnGen Consortium (3) | AUD | 13,422 | 244,533 | data available |
| FinnGen Consortium (3) | OUD | 775 | 255,921 | data available |
| PGC Substance Use Disorders working group, iPSYCH, and deCODE (4) | CUD | 14,080 | 343,726 | data available |

# Table S2 Summary of single and common factor GWAS results

| Model | SNP-h^2^ | lambda | number of hits  (5x10^-08^) | Q_SNP_ hits | mean chi^2^ | LDSC intercept |
| --- | --- | --- | --- | --- | --- | --- |
| Externalizing factor  (EXT) | 0.06 | 1.91 | 344 | 4 | 2.37 | 0.98 (0.02) |
| Externalizing GWAS  from Externalizing Consortium (EXT-CON) | 0.10 | 1.41 | 579 | 160 | 1.45 | 1.26 (0.01) |
| ADHD | 0.23 | 1.25 | 317 |  | 1.29 | 1.03 (0.01) |
| CD | 0.01 | 1.03 | 0 |  | 1.02 | 1.00 (0.01) |
| ASPD | 0.01 | 1.02 | 0 |  | 1.02 | 0.99 (0.01) |
| AUD | 0.02 | 1.15 | 83 |  | 1.17 | 1.07 (0.01) |
| OUD | 0.01 | 1.01 | 1 |  | 1.01 | 0.99 (0.01) |
| CUD | 0.20 | 1.11 | 29 |  | 1.11 | 0.99 (0.01) |

Notes. *, ADHD = Attention deficit hyperactivity disorder, CD = Conduct disorder, ASPD = antisocial personality disorder, AUD = Alcohol use disorder, OUD = Opioid use disorder, CUD = Cannabinoid use disorder, AIC = Akaike information criterion, CFI = comparative fit index, SRMR = standardized root mean square residual.*

# Table S3 Results of the MAGMA gene-based association analysis after after Bonferroni correction (defined at 2.61x10^-6^)

| GENE | CHR | START | STOP | NSNPS | NPARAM | N | ZSTAT | P | SYMBOL |
| --- | --- | --- | --- | --- | --- | --- | --- | --- | --- |
| ENSG00000128573 | 7 | 113676382 | 114383827 | 833 | 47 | 80522 | 6.5044 | 3.90E-11 | FOXP2 |
| ENSG00000175161 | 3 | 84958132 | 86173579 | 2829 | 55 | 80522 | 6.2789 | 1.70E-10 | CADM2 |
| ENSG00000152208 | 4 | 93175550 | 94745707 | 2983 | 102 | 80522 | 5.8372 | 2.66E-09 | GRID2 |
| ENSG00000164061 | 3 | 49541922 | 49758978 | 258 | 13 | 80522 | 5.6586 | 7.63E-09 | BSN |
| ENSG00000137872 | 15 | 47426298 | 48116420 | 1627 | 63 | 80522 | 5.5916 | 1.12E-08 | SEMA6D |
| ENSG00000174442 | 15 | 66747297 | 66892115 | 415 | 32 | 80522 | 5.4741 | 2.20E-08 | ZWILCH |
| ENSG00000114316 | 3 | 49265264 | 49428145 | 177 | 16 | 80522 | 5.4683 | 2.27E-08 | USP4 |
| ENSG00000185909 | 3 | 49159044 | 49263917 | 103 | 10 | 80522 | 5.4353 | 2.74E-08 | KLHDC8B |
| ENSG00000236980 | 3 | 49165065 | 49279291 | 125 | 11 | 80522 | 5.4229 | 2.93E-08 | C3orf84 |
| ENSG00000177352 | 3 | 49149968 | 49253754 | 101 | 10 | 80522 | 5.3945 | 3.44E-08 | CCDC71 |
| ENSG00000188315 | 3 | 49256035 | 49365342 | 108 | 12 | 80522 | 5.382 | 3.68E-08 | C3orf62 |
| ENSG00000173421 | 3 | 49185861 | 49345537 | 168 | 14 | 80522 | 5.3655 | 4.04E-08 | CCDC36 |
| ENSG00000188501 | 15 | 66789517 | 66908317 | 354 | 33 | 80522 | 5.3116 | 5.43E-08 | LCTL |
| ENSG00000233276 | 3 | 49344609 | 49446033 | 122 | 11 | 80522 | 5.2435 | 7.88E-08 | GPX1 |
| ENSG00000169744 | 4 | 16453164 | 16950432 | 990 | 93 | 80522 | 5.2112 | 9.38E-08 | LDB2 |
| ENSG00000172037 | 3 | 49108547 | 49220551 | 101 | 11 | 80522 | 5.2083 | 9.53E-08 | LAMB2 |
| ENSG00000225399 | 3 | 49247518 | 49348744 | 106 | 12 | 80522 | 5.1819 | 1.10E-07 | RP11-3B7.1 |
| ENSG00000138640 | 4 | 89597106 | 90082549 | 1095 | 49 | 80522 | 5.127 | 1.47E-07 | FAM13A |
| ENSG00000120885 | 8 | 27404434 | 27522548 | 318 | 33 | 80522 | 5.1139 | 1.58E-07 | CLU |
| ENSG00000187323 | 18 | 49816542 | 51107784 | 4131 | 99 | 80522 | 5.0924 | 1.77E-07 | DCC |
| ENSG00000164062 | 3 | 49661435 | 49771396 | 133 | 12 | 80522 | 5.0841 | 1.85E-07 | APEH |
| ENSG00000139318 | 12 | 89691009 | 89797048 | 194 | 25 | 80522 | 5.0836 | 1.85E-07 | DUSP6 |
| ENSG00000172046 | 3 | 49095479 | 49208371 | 88 | 10 | 80522 | 5.0546 | 2.16E-07 | USP19 |
| ENSG00000067560 | 3 | 49346578 | 49500431 | 181 | 13 | 80522 | 5.024 | 2.53E-07 | RHOA |
| ENSG00000175497 | 2 | 115149876 | 116653328 | 3032 | 110 | 80522 | 4.9889 | 3.04E-07 | DPP10 |
| ENSG00000259471 | 15 | 66824528 | 67028132 | 577 | 53 | 80522 | 4.9816 | 3.15E-07 | RP11-321F6.1 |
| ENSG00000156564 | 6 | 40309325 | 40605204 | 941 | 55 | 80522 | 4.9762 | 3.24E-07 | LRFN2 |
| ENSG00000174444 | 15 | 66740355 | 66866870 | 325 | 22 | 80522 | 4.957 | 3.58E-07 | RPL4 |
| ENSG00000172053 | 3 | 49083365 | 49192553 | 79 | 9 | 80522 | 4.929 | 4.13E-07 | QARS |
| ENSG00000173531 | 3 | 49671380 | 49776934 | 129 | 12 | 80522 | 4.9165 | 4.41E-07 | MST1 |
| ENSG00000196782 | 4 | 140587907 | 141125338 | 940 | 80 | 80522 | 4.9078 | 4.61E-07 | MAML3 |
| ENSG00000173402 | 3 | 49456146 | 49623048 | 193 | 13 | 80522 | 4.8747 | 5.45E-07 | DAG1 |
| ENSG00000198218 | 3 | 49017140 | 49181796 | 103 | 12 | 80522 | 4.8267 | 6.94E-07 | QRICH1 |
| ENSG00000143641 | 1 | 230143536 | 230467870 | 917 | 79 | 80522 | 4.8091 | 7.58E-07 | GALNT2 |
| ENSG00000166938 | 15 | 66535555 | 66676236 | 304 | 23 | 80522 | 4.7976 | 8.03E-07 | DIS3L |
| ENSG00000116329 | 1 | 29088654 | 29240208 | 352 | 33 | 80522 | 4.7947 | 8.15E-07 | OPRD1 |
| ENSG00000174446 | 15 | 66732473 | 66840151 | 257 | 17 | 80522 | 4.7457 | 1.04E-06 | SNAPC5 |
| ENSG00000079102 | 8 | 92917203 | 93165514 | 228 | 10 | 80522 | 4.7292 | 1.13E-06 | RUNX1T1 |
| ENSG00000143032 | 1 | 91127096 | 91232794 | 249 | 31 | 80522 | 4.7068 | 1.26E-06 | BARHL2 |
| ENSG00000145022 | 3 | 49399639 | 49503908 | 129 | 10 | 80522 | 4.7034 | 1.28E-06 | TCTA |
| ENSG00000169032 | 15 | 66629155 | 66834650 | 483 | 22 | 80522 | 4.6237 | 1.88E-06 | MAP2K1 |
| ENSG00000145029 | 3 | 49410379 | 49516759 | 128 | 10 | 80522 | 4.6033 | 2.08E-06 | NICN1 |
| ENSG00000178035 | 3 | 49011758 | 49116841 | 65 | 8 | 80522 | 4.5793 | 2.33E-06 | IMPDH2 |
| ENSG00000178467 | 3 | 48977319 | 49094587 | 75 | 9 | 80522 | 4.5685 | 2.46E-06 | P4HTM |
| ENSG00000178057 | 3 | 49007892 | 49110928 | 60 | 7 | 80522 | 4.5664 | 2.48E-06 | NDUFAF3 |
| ENSG00000075131 | 15 | 66578544 | 66729084 | 339 | 21 | 80522 | 4.5649 | 2.50E-06 | TIPIN |
| ENSG00000145020 | 3 | 49404211 | 49510186 | 129 | 10 | 80522 | 4.5641 | 2.51E-06 | AMT |
| ENSG00000178149 | 3 | 49002921 | 49109726 | 60 | 7 | 80522 | 4.5628 | 2.52E-06 | DALRD3 |
| ENSG00000164796 | 8 | 113185157 | 114499328 | 2705 | 65 | 80522 | 4.5598 | 2.56E-06 | CSMD3 |

# Table S4 Power analyses for Mendelian Randomization analysis with GWAS for childhood maltreatment from UK Biobank

|  | ADHD | CD | ASPD | AUD | OUD | CUD |
| --- | --- | --- | --- | --- | --- | --- |
| *OR*=1.10 | 0.07 | 0.05 | 0.05 | 0.07 | 0.051 | 0.074 |
| *OR*=1.20 | 0.13 | 0.05 | 0.05 | 0.14 | 0.056 | 0.149 |
| *OR*=1.30 | 0.23 | 0.06 | 0.06 | 0.26 | 0.062 | 0.275 |
| *OR*=1.40 | 0.35 | 0.06 | 0.06 | 0.42 | 0.072 | 0.441 |
| *OR*=1.50 | 0.49 | 0.07 | 0.07 | 0.59 | 0.085 | 0.617 |
| *OR*=1.60 | 0.62 | 0.08 | 0.08 | 0.74 | 0.101 | 0.772 |
| *OR*=1.70 | 0.73 | 0.09 | 0.08 | 0.86 | 0.119 | 0.883 |
| *OR*=1.80 | 0.83 | 0.10 | 0.09 | **0.93** | 0.141 | **0.949** |
| *OR*=1.90 | 0.89 | 0.12 | 0.11 | 0.97 | 0.166 | 0.981 |
| *OR*=2.00 | **0.94** | 0.13 | 0.12 | 0.99 | 0.194 | 0.994 |
| *OR*=4.00 | 1.00 | 0.71 | 0.64 | 1.00 | **0.91** | 1.00 |
| *OR*=4.50 | 1.00 | 0.84 | 0.77 | 1.00 | 0.972 | 1.00 |
| *OR*=5.00 | 1.00 | **0.92** | 0.87 | 1.00 | 0.993 | 1.00 |
| *N* total | 55,374 | 257,310 | 253,258 | 257,955 | 256,696 | 357,806 |
| Cases % | 0.36 | < 0.01 | < 0.01 | 0.05 | < 0.01 | 0.04 |

Note. ADHD = Attention deficit hyperactivity disorder, CD = Conduct disorder, ASPD = antisocial personality disorder, AUD = Alcohol use disorder, OUD = Opioid use disorder, CUD = Cannabinoid use disorder.

# Table S5 Associations of single nucleotide polymorphisms with childhood maltreatment and ADHD, CD, ASPD, AUD, OUD, CUD, and the externalizing factor

| Exposure/outcome | SNP | beta | SE | P-value | R^2^ | F-value |
| --- | --- | --- | --- | --- | --- | --- |
| Childhood | rs13090329 | 0.0156387 | 0.0028581 | 4.60E-08 | 0.00021 | 29.94 |
| maltreatment | rs611531 | -0.0175435 | 0.00321086 | 4.60E-08 | 0.00021 | 29.85 |
|  | rs1015511 | -0.0173112 | 0.00271547 | 1.90E-10 | 0.00028 | 40.64 |
|  | rs3843947 | -0.0168222 | 0.00270854 | 5.00E-10 | 0.00027 | 38.57 |
|  | rs1350269 | -0.0148856 | 0.00270749 | 4.40E-08 | 0.00021 | 30.23 |
|  | rs4702 | 0.0195476 | 0.0026698 | 2.30E-13 | 0.00037 | 53.61 |
| ADHD | rs13090329 | 0.013 | 0.017 | 0.4399 |  |  |
|  | rs611531 | -0.026 | 0.017 | 0.1187 |  |  |
|  | rs1015511 | -0.065 | 0.014 | 2.591E-06 |  |  |
|  | rs3843947 | -0.039 | 0.014 | 0.004805 |  |  |
|  | rs1350269 | -0.036 | 0.014 | 0.008932 |  |  |
|  | rs4702 | 0.043 | 0.014 | 0.002325 |  |  |
| CD | rs13090329 | -0.086 | 0.075 | 0.2500862 |  |  |
|  | rs611531 | -0.049 | 0.073 | 0.5045751 |  |  |
|  | rs1015511 | -0.091 | 0.069 | 0.1848967 |  |  |
|  | rs3843947 | -0.042 | 0.068 | 0.5387234 |  |  |
|  | rs1350269 | -0.022 | 0.068 | 0.7440748 |  |  |
|  | rs4702 | 0.129 | 0.068 | 0.0596423 |  |  |
| ASPD | rs13090329 | -0.058 | 0.082 | 0.4813425 |  |  |
|  | rs611531 | 0.086 | 0.079 | 0.2787539 |  |  |
|  | rs1015511 | -0.072 | 0.075 | 0.338645 |  |  |
|  | rs3843947 | -0.015 | 0.075 | 0.8450253 |  |  |
|  | rs1350269 | -0.07 | 0.075 | 0.3475784 |  |  |
|  | rs4702 | 0.135 | 0.075 | 0.0705735 |  |  |
| AUD | rs13090329 | 0.004 | 0.015 | 0.8097842 |  |  |
|  | rs611531 | -0.017 | 0.015 | 0.2527795 |  |  |
|  | rs1015511 | -0.042 | 0.014 | 0.0028882 |  |  |
|  | rs3843947 | -0.011 | 0.014 | 0.4419196 |  |  |
|  | rs1350269 | -0.011 | 0.014 | 0.4242871 |  |  |
|  | rs4702 | 0.041 | 0.014 | 0.0033757 |  |  |
| OUD | rs13090329 | 0.046 | 0.058 | 0.4196178 |  |  |
|  | rs611531 | 0.015 | 0.056 | 0.7834608 |  |  |
|  | rs1015511 | -0.162 | 0.053 | 0.0022165 |  |  |
|  | rs3843947 | 0.051 | 0.053 | 0.3284726 |  |  |
|  | rs1350269 | -0.018 | 0.053 | 0.735346 |  |  |
|  | rs4702 | 0.073 | 0.052 | 0.1617423 |  |  |
| CUD | rs13090329 | 0.043 | 0.02 | 0.03237 |  |  |
|  | rs611531 | -0.021 | 0.02 | 0.3042 |  |  |
|  | rs1015511 | -0.096 | 0.017 | 1.3E-08 |  |  |
|  | rs3843947 | 0.021 | 0.017 | 0.2171 |  |  |
|  | rs1350269 | -0.005 | 0.017 | 0.7836 |  |  |
|  | rs4702 | 0.041 | 0.017 | 0.0154 |  |  |
| Externalizing factor | rs13090329 | 0.01 | 0.006 | 0.1308834 |  |  |
| (EXT) | rs611531 | -0.011 | 0.007 | 0.0949376 |  |  |
|  | rs1015511 | -0.042 | 0.006 | 7.714E-13 |  |  |
|  | rs3843947 | -0.007 | 0.006 | 0.2384789 |  |  |
|  | rs1350269 | -0.016 | 0.006 | 0.0047852 |  |  |
|  | rs4702 | 0.027 | 0.006 | 3.307E-06 |  |  |

Note. ADHD = Attention deficit hyperactivity disorder, CD = Conduct disorder, ASPD = antisocial personality disorder, AUD = Alcohol use disorder, OUD = Opioid use disorder, CUD = Cannabinoid use disorder.

# Table S6 Mendelian Randomization estimates for association between childhood maltreatment and ADHD, CD, ASPD, AUD, OUD, CUD as well as the externalizing factor

|  |  |  | Instrumental variables (IVs) (*P* < 5 x 10^–08^) | | | |
| --- | --- | --- | --- | --- | --- | --- |
| Outcome | method | *N* | *OR* | *CI* | *P*-Value | q-value |
| ADHD | IVW | 6 | 10.09 | (4.76-21.40) | 1.63x10^-09^ | 2.96x10^-08^ |
|  | IVW radial | 6 | 10.01 | (4.71-21.27) | 2.11x10^-09^ | 2.96x10^-08^ |
|  | Weighted median | 6 | 9.57 | (3.50-26.17) | 1.08x10^-05^ | 1.01x10^-04^ |
|  | MR PRESSO | 6 | 10.09 | (4.76-21.40) | 0.002 | 0.006 |
| CD | IVW | 6 | 17.52 | (0.65-471.38) | 0.088 | 0.137 |
|  | IVW radial | 6 | 17.51 | (0.73-419.87) | 0.077 | 0.127 |
|  | Weighted median | 6 | 15.49 | (0.23-1025.34) | 0.200 | 0.237 |
|  | MR PRESSO | 6 | 17.52 | (0.73-419.44) | 0.137 | 0.203 |
| ASPD | IVW | 6 | 6.39 | (0.14-303.57) | 0.346 | 0.359 |
|  | IVW radial | 6 | 6.40 | (0.14-303.80) | 0.346 | 0.359 |
|  | Weighted median | 6 | 24.16 | (0.19-3162.44) | 0.200 | 0.237 |
|  | MR PRESSO | 6 | 6.39 | (0.14-303.57) | 0.390 | 0.390 |
| AUD | IVW | 6 | 3.72 | (1.85-7.52) | 2.42x10^-04^ | 0.001 |
|  | IVW radial | 6 | 3.73 | (1.85-7.53) | 2.45x10^-04^ | 0.001 |
|  | Weighted median | 6 | 2.50 | (0.97-6.44) | 0.058 | 0.105 |
|  | MR PRESSO | 6 | 3.72 | (1.85-7.52) | 0.014 | 0.040 |
| OUD | IVW | 6 | 10.97 | (0.33-368.22) | 0.182 | 0.237 |
|  | IVW radial | 6 | 10.99 | (0.33-369.07) | 0.181 | 0.237 |
|  | Weighted median | 6 | 10.10 | (0.29-356.32) | 0.203 | 0.237 |
|  | MR PRESSO | 6 | 10.97 | (0.33-368.22) | 0.239 | 0.268 |
| CUD | IVW | 6 | 6.29 | (0.93-42.75) | 0.060 | 0.105 |
|  | IVW radial | 6 | 6.30 | (0.93-42.71) | 0.059 | 0.105 |
|  | Weighted median | 6 | 4.30 | (1.20-15.43) | 0.025 | 0.059 |
|  | MR PRESSO | 4 | 4.97 | (1.89-13.08) | 0.048 | 0.102 |
| Externalizing factor (EXT) | IVW | 6 | 2.64 | (1.52-4.60) | 5.80x10^-04^ | 0.002 |
|  | IVW radial | 6 | 2.67 | (1.52-4.66) | 5.71x10^-04^ | 0.002 |
|  | Weighted median | 6 | 1.71 | (1.13-2.59) | 0.010 | 0.033 |
|  | MR PRESSO | 5 | 2.13 | (1.44-3.14) | 0.020 | 0.049 |

Notes. *OR* = odds ratio, *CI* = confidence interval, IVW = Inverse variance weighted, MR PRESSO = Mendelian Randomization Pleiotropy RESidual Sum and Outlier, ADHD = Attention deficit hyperactivity disorder, CD = Conduct disorder, ASPD = antisocial personality disorder, AUD = Alcohol use disorder, OUD = Opioid use disorder, CUD = Cannabinoid use disorder.

# Table S7 Heterogeneity of Wald ratios and MR-Egger test for directional pleiotropy

| outcome | Heterogeneity | | | |  |  |
| --- | --- | --- | --- | --- | --- | --- |
|  | Instrumental variables (*P*<5x10^-8^) | | | |  |  |
|  | *Q* | *df* | *I^2^* | *p* value |  |  |
| ADHD | 5.96 | 5 | 0.16 | 0.310 |  |  |
| CD | 4.65 | 5 | 0.07 | 0.460 |  |  |
| ASPD | 5.75 | 5 | 0.13 | 0.331 |  |  |
| AUD | 5.43 | 5 | 0.08 | 0.366 |  |  |
| OUD | 9.67 | 5 | 0.48 | 0.085 |  |  |
| CUD | 26.65 | 5 | 0.81 | 6.70x10^-05^ |  |  |
| Externalizing factor  (EXT) | 23.68 | 5 | 0.79 | 2.50x10^-04^ |  |  |
|  |  | Intercept | se | *p* value | *OR* | *CI* |
| ADHD |  | -0.0210 | 0.0809 | 0.795 | 34.44 | (0.00-379815.84) |
| CD |  | -0.5173 | 0.3247 | 0.111 | 2.34x10^+14^ | (0.01-3.81x10^+30^) |
| ASPD |  | -0.2889 | 0.4009 | 0.471 | 136606360 | (0-1.40x10^+28^) |
| AUD |  | -0.1072 | 0.0666 | 0.107 | 1945.584 | (0.93-4080339.28) |
| OUD |  | -0.1600 | 0.3790 | 0.673 | 125674.2 | (0-1.04x10^+24^) |
| CUD |  | -0.0988 | 0.2018 | 0.625 | 2019.38 | (0-2.42x10^+13^) |
| Externalizing factor  (EXT) |  | -0.0483 | 0.0545 | 0.375 | 44.69 | (0.08-23658.02) |

Notes. SNP = Single nucleotide polymorphism, *Q* = heterogeneity statistic Q, *df* = degree of freedom, *se* = standard error, ADHD = Attention deficit hyperactivity disorder, CD = Conduct disorder, ASPD = antisocial personality disorder, AUD = Alcohol use disorder, OUD = Opioid use disorder, CUD = Cannabinoid use disorder.

# Table S8 Leave-one-out analysis

| outcome | SNP | *OR* | *CI* | *P-Value* |
| --- | --- | --- | --- | --- |
| ADHD | rs1015511 | 7.12 | 3.31-15.33 | 5.09E-07 |
|  | rs13090329 | 12.02 | 5.81-24.87 | 2.081E-11 |
|  | rs1350269 | 9.96 | 4.01-24.71 | 7.26E-07 |
|  | rs3843947 | 10.05 | 3.98-25.42 | 1.088E-06 |
|  | rs4702 | 10.47 | 4.01-27.3 | 1.581E-06 |
|  | rs611531 | 11.49 | 4.98-26.49 | 1.014E-08 |
|  | All | 10.09 | 4.76-21.4 | 1.631E-09 |
| Conduct disorder | rs1015511 | 10.38 | 0.25-430.27 | 0.2180689 |
|  | rs13090329 | 56.65 | 1.69-1904.88 | 0.0244012 |
|  | rs1350269 | 21.62 | 0.5-940.22 | 0.1102754 |
|  | rs3843947 | 18.86 | 0.38-925.83 | 0.1392325 |
|  | rs4702 | 5.74 | 0.13-244.8 | 0.3616231 |
|  | rs611531 | 17.86 | 0.37-867.76 | 0.1456897 |
|  | All | 17.52 | 0.65-471.38 | 0.0882508 |
| ASPD | rs1015511 | 3.87 | 0.04-393.39 | 0.5658003 |
|  | rs13090329 | 13.82 | 0.24-805.48 | 0.2054525 |
|  | rs1350269 | 4.11 | 0.05-363.89 | 0.5366135 |
|  | rs3843947 | 7.81 | 0.07-868.99 | 0.3925519 |
|  | rs4702 | 1.40 | 0.02-84.93 | 0.8711555 |
|  | rs611531 | 24.36 | 0.47-1252.27 | 0.1121756 |
|  | All | 6.39 | 0.14-303.57 | 0.3463142 |
| AUD | rs1015511 | 2.93 | 1.39-6.16 | 0.0046399 |
|  | rs13090329 | 4.33 | 2.1-8.94 | 7.189E-05 |
|  | rs1350269 | 4.06 | 1.8-9.12 | 0.0007138 |
|  | rs3843947 | 4.28 | 1.93-9.49 | 0.0003444 |
|  | rs4702 | 2.95 | 1.37-6.35 | 0.0058661 |
|  | rs611531 | 3.98 | 1.72-9.25 | 0.0013083 |
|  | All | 3.72 | 1.85-7.52 | 0.0002421 |
| OD | rs1015511 | 2.43 | 0.15-39.46 | 0.5327704 |
|  | rs13090329 | 10.12 | 0.15-666.6 | 0.2788166 |
|  | rs1350269 | 13.19 | 0.2-870.99 | 0.2276848 |
|  | rs3843947 | 33.47 | 1.12-1002.29 | 0.0429398 |
|  | rs4702 | 7.29 | 0.09-596.69 | 0.3767028 |
|  | rs611531 | 20.85 | 0.38-1145.87 | 0.137355 |
|  | All | 10.97 | 0.33-368.22 | 0.1816249 |
| CUD | rs1015511 | 2.65 | 0.67-10.44 | 0.1647871 |
|  | rs13090329 | 5.63 | 0.59-53.43 | 0.1321581 |
|  | rs1350269 | 8.14 | 0.89-74.82 | 0.0639109 |
|  | rs3843947 | 12.34 | 2.08-73.19 | 0.0056492 |
|  | rs4702 | 5.81 | 0.5-67.44 | 0.1596794 |
|  | rs611531 | 6.95 | 0.71-68.41 | 0.0964251 |
|  | All | 6.29 | 0.93-42.75 | 0.0599525 |
| Externalizing factor | rs1015511 | 2.38 | 1.63-3.46 | 6.258E-06 |
| (EXT) | rs13090329 | 3.37 | 1.69-6.7 | 0.0005509 |
|  | rs1350269 | 3.18 | 1.54-6.56 | 0.0017998 |
|  | rs3843947 | 3.69 | 1.94-7 | 6.493E-05 |
|  | rs4702 | 2.93 | 1.37-6.23 | 0.0053619 |
|  | rs611531 | 3.43 | 1.72-6.84 | 0.0004824 |
|  | All | 3.14 | 1.72-5.73 | 0.0001968 |

Notes. SNP = Single nucleotide polymorphism, *OR* = odds ratio, *CI* = confidence interval, ADHD = Attention deficit hyperactivity disorder, CD = Conduct disorder, ASPD = antisocial personality disorder, AUD = Alcohol use disorder, OUD = Opioid use disorder, CUD = Cannabinoid use disorder.

Table S9 Causal Analysis Using Summary Effect estimates (CAUSE) for association between prospective childhood maltreatment on ADHD, CD, ASPD, AUD, OUD, CUD as well as the externalizing factor

|  |  | Causal estimation | | |  |
| --- | --- | --- | --- | --- | --- |
| Exposure | Cancer site | *OR* | *CredIn* | *P*-Value |  |
| prospective childhood maltreatment | ADHD | 1.22 | (0.66-2.23) | 0.514 |  |
|  | CD | 1.00 | (0.00-1.19x10^6^) | 1.000 |  |
|  | ASPD | 0.01 | (0.00-1.25) | 0.494 |  |
|  | AUD | 1.77 | (0.65-4.35) | 0.214 |  |
|  | OUD | 1.00 | (0.00-953.37) | 1.000 |  |
|  | CUD | 1.39 | (0.66-2.92) | 0.382 |  |
|  | Externalizing factor (EXT) | 1.31 | (1.04-1.64) | 0.021 |  |

Notes. *OR* = odds ratio, C*redIn* = credible interval, ADHD = Attention deficit hyperactivity disorder, CD = Conduct disorder, ASPD = antisocial personality disorder, AUD = Alcohol use disorder, OUD = Opioid use disorder, CUD = Cannabinoid use disorder.


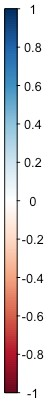


|  | **ADHD** | **CD** | **ASPD** | **AUD** | **OUD** | **CUD** |
| --- | --- | --- | --- | --- | --- | --- |
| **1** | **1.00** | **0.50**  **(0.11)** | **0.51**  **(0.09)** | **0.50**  **(0.06)** | **0.57**  **(0.12)** | **0.52**  **(0.06)** |
| **2** |  | **1.00** | **0.68**  **(0.26)** | **0.60**  **(0.16)** | **0.86**  **(0.33)** | **0.52**  **(0.13)** |
| **3** |  |  | **1.00** | **0.87**  **(0.14)** | **0.21**  **(0.29)** | **0.58**  **(0.12)** |
| **4** |  |  |  | **1.00** | **0.86**  **(0.17)** | **0.71**  **(0.07)** |
| **5** |  |  |  |  | **1.00** | **0.71**  **(0.14)** |
| **6** |  |  |  |  |  | **1.00** |

# *Supplementary Figure S1*. Genetic correlation matrix of attention deficit hyperactivity disorder (ADHD), conduct disorder (CD), antisocial personality disorder (ASPD), alcohol use disorder (AUD), opioid use disorder (OUD) and cannabinoid use disorder (CUD).


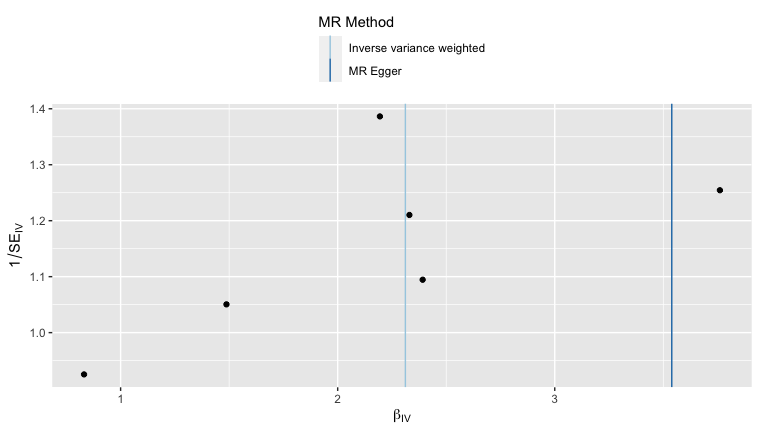

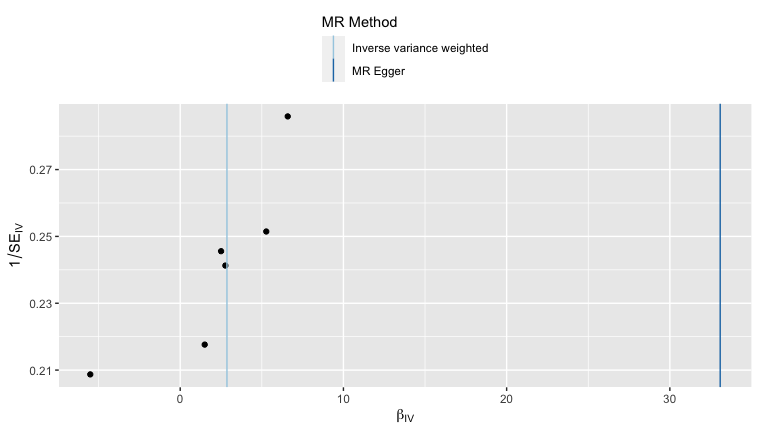


B

A


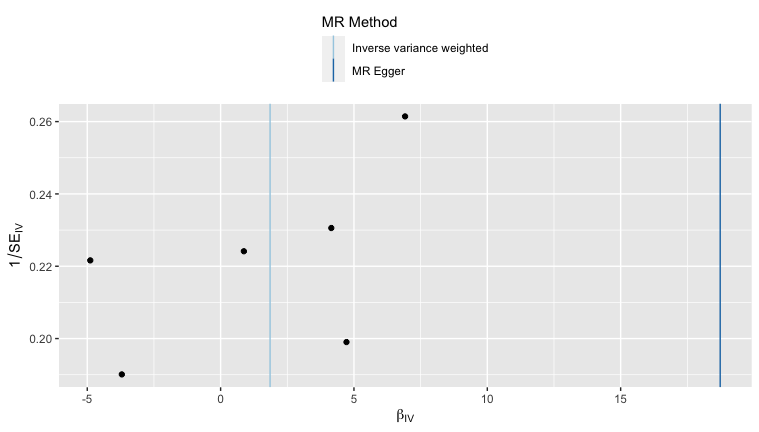

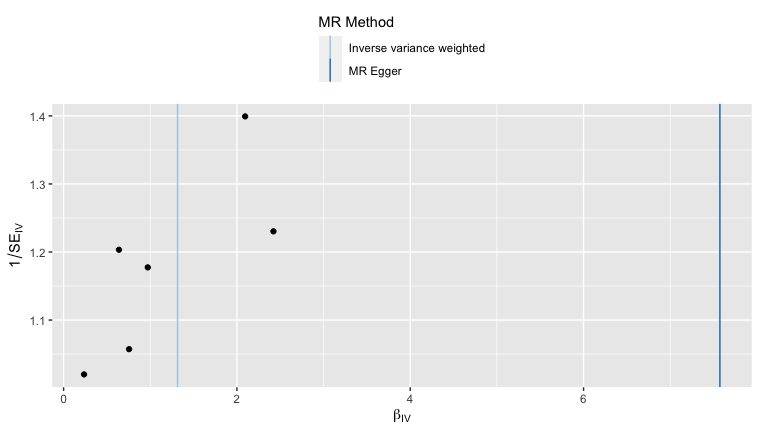


C

D


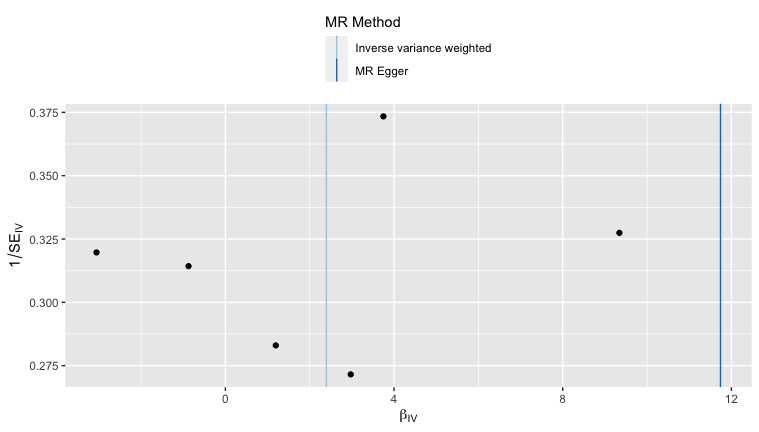

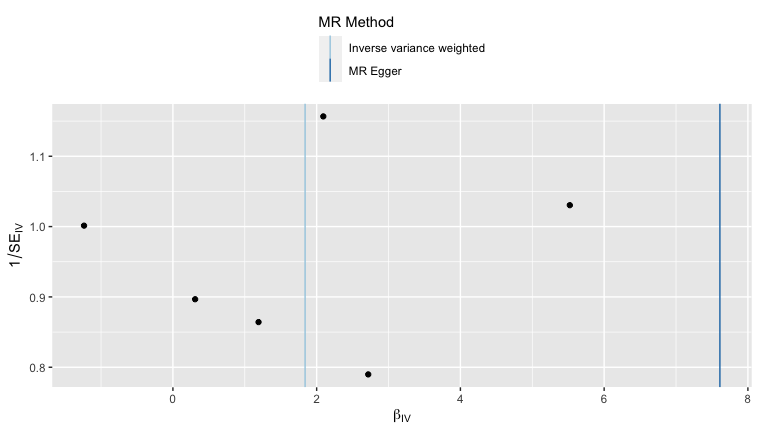

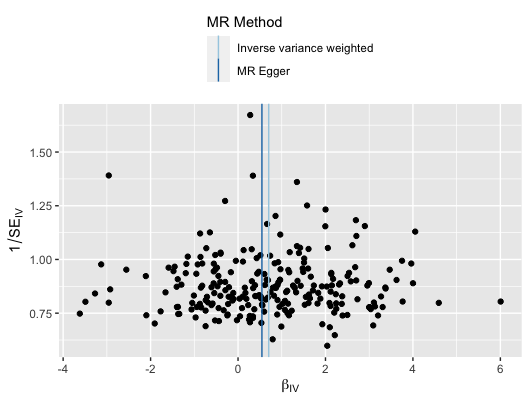


E

F


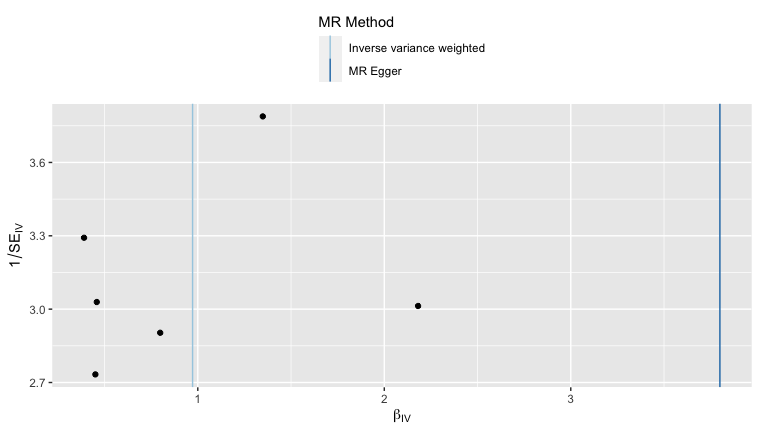


G

# *Supplementary Figure S2*. Funnel plots showing relationship between the SNP associations with childhood maltreatment and the SNP associations with (A) attention deficit hyperactivity disorder, (B) associations with conduct disorder, (C) associations with antisocial personality disorder, (D) associations with alcohol use disorder, (E) associations with opioid use disorder, (F) associations with cannabinoid use disorder and SNP associations with the (G) externalizing factor.

*
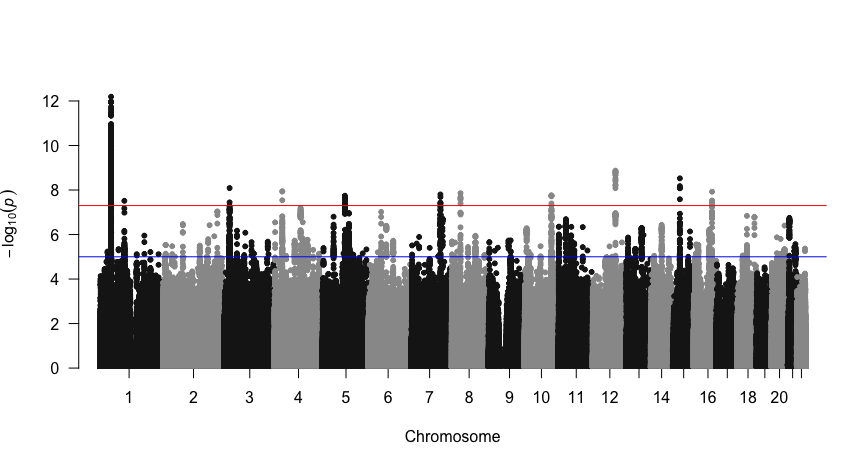
*

ADHD

*
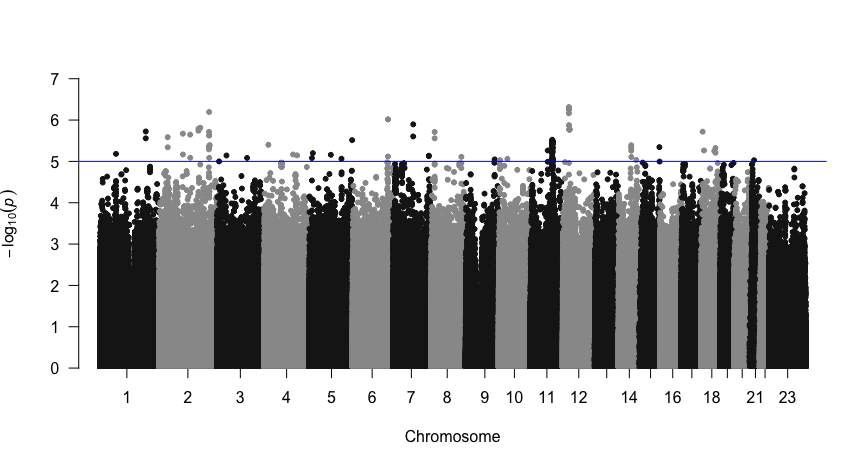
*

CD

*
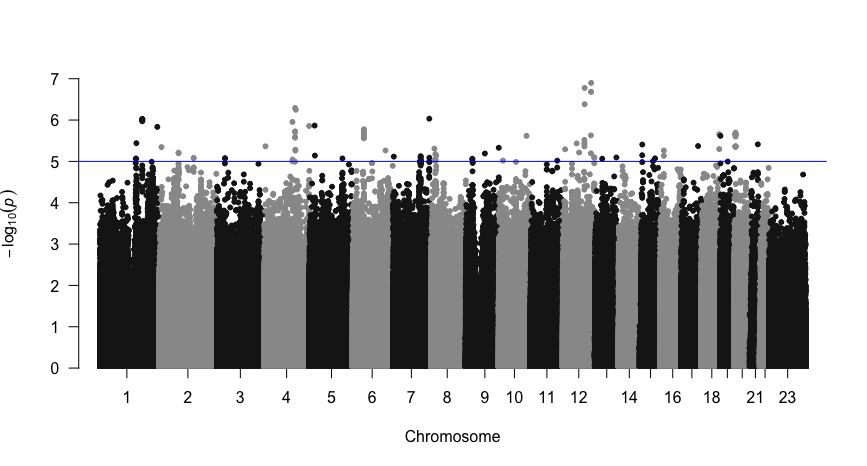
*

ASPD

*
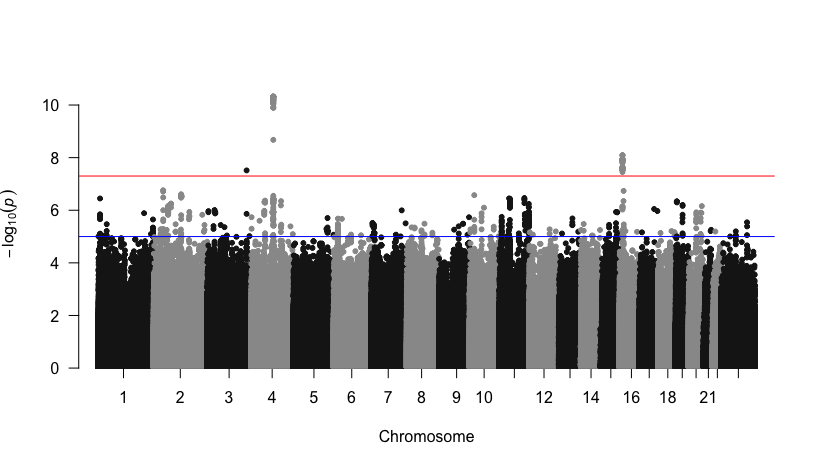
*

AUD

*
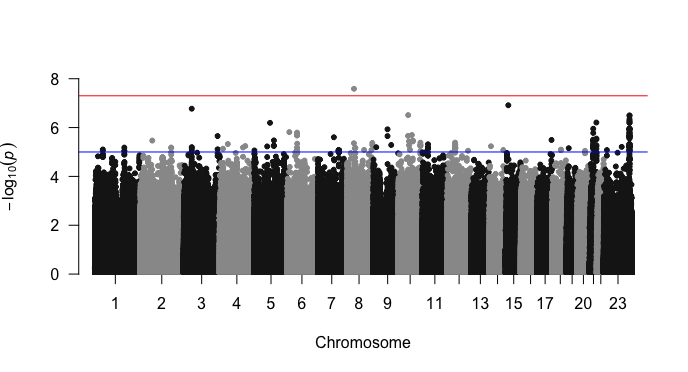
*

OUD

*
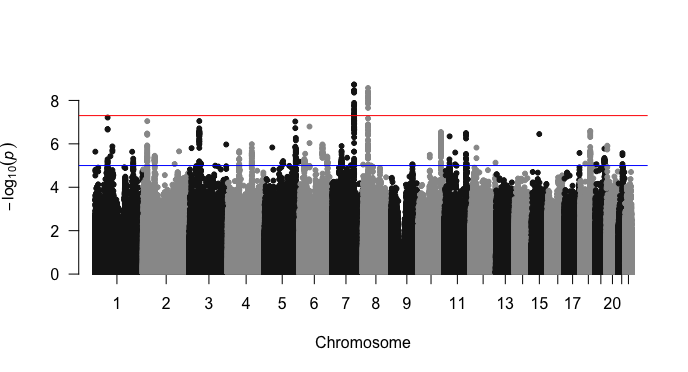
*

CUD

# Supplementary Figure S3. Manhattan plots of GWAS for attention deficit hyperactivity disorder (ADHD), conduct disorder (CD), antisocial personality disorder (ASPD), alcohol use disorder (AUD), opioid use disorder (OUD), and cannabinoid use disorder (CUD).


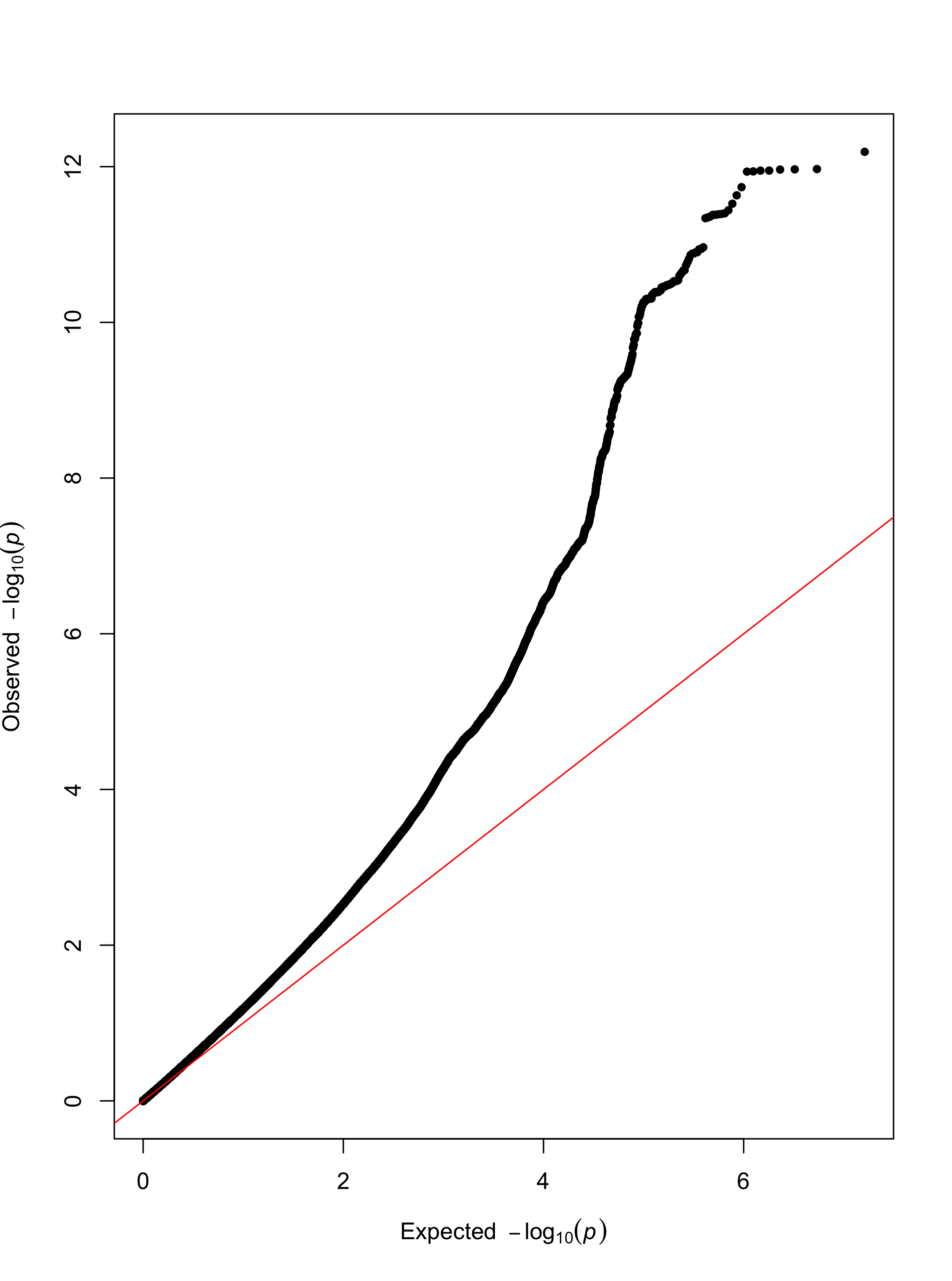

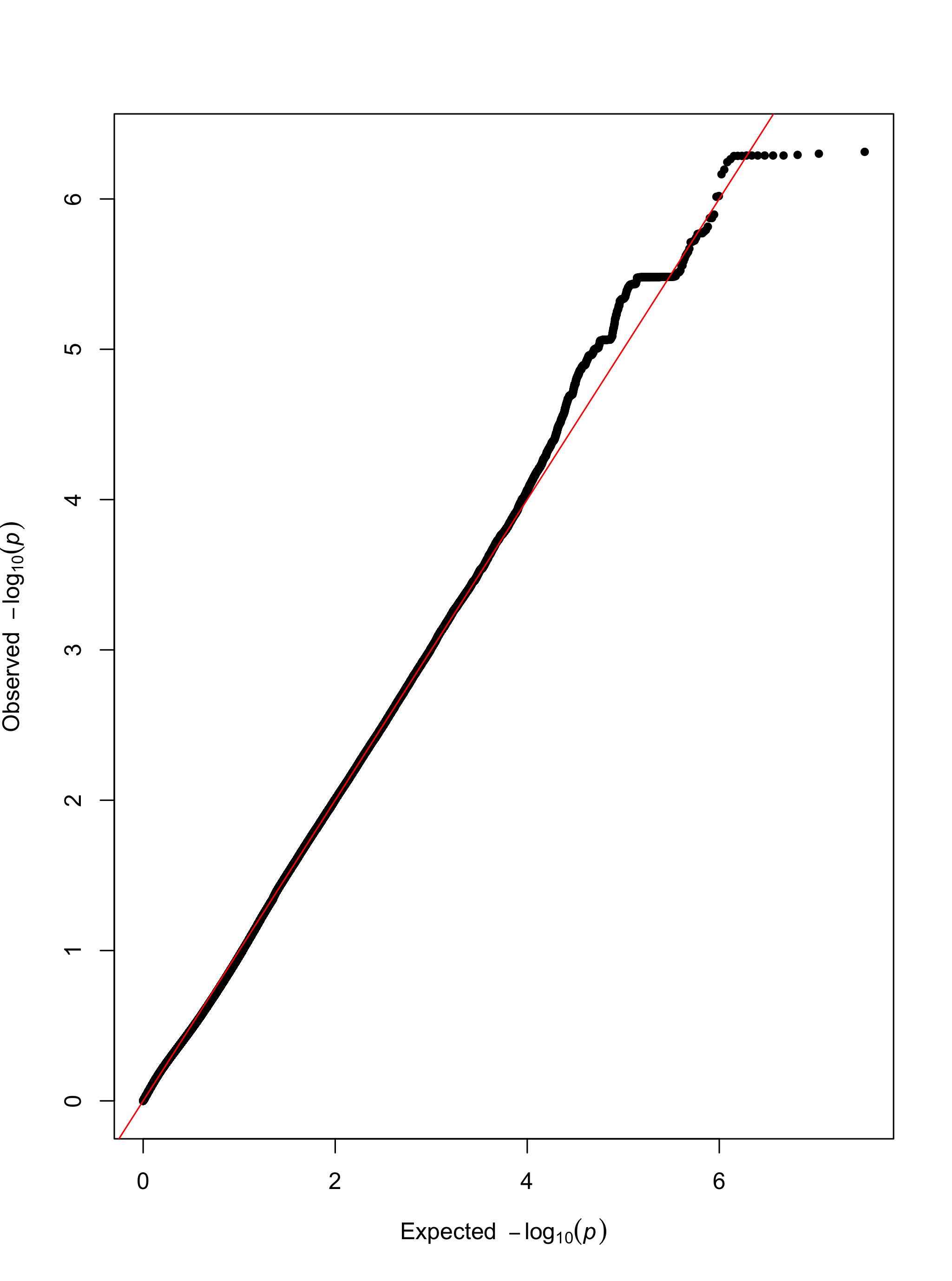


ADHD

CD


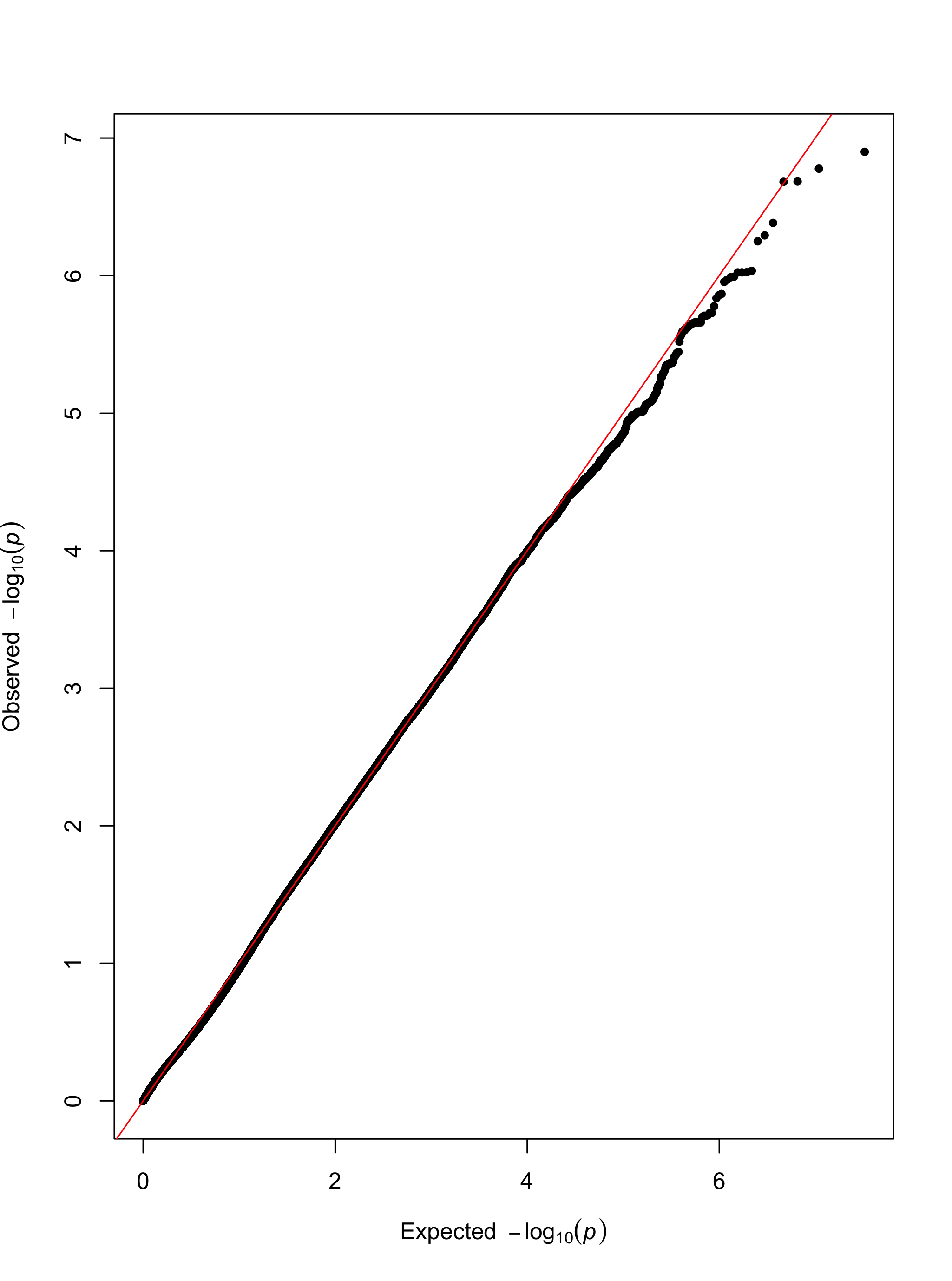

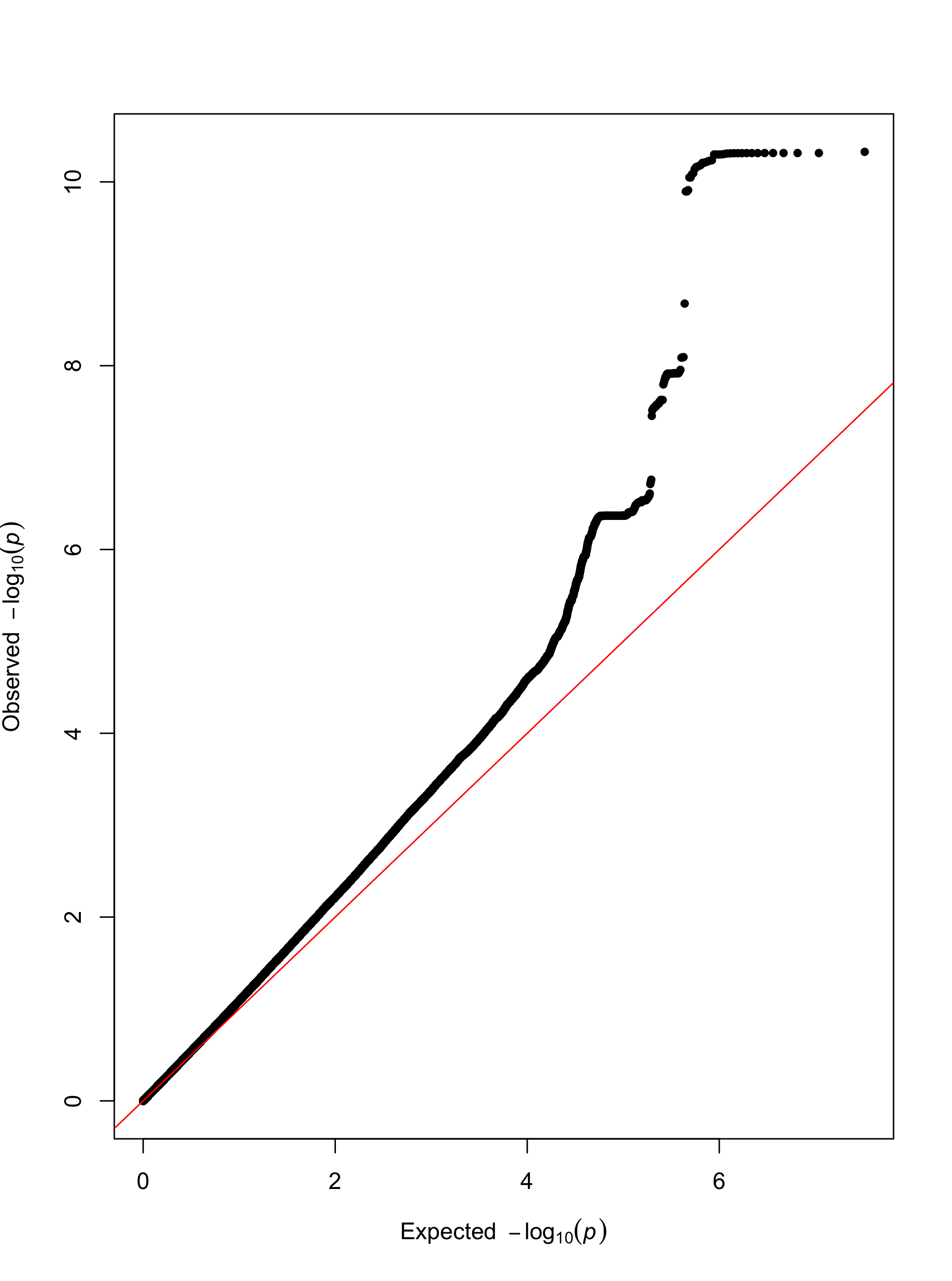


ASPD

AUD


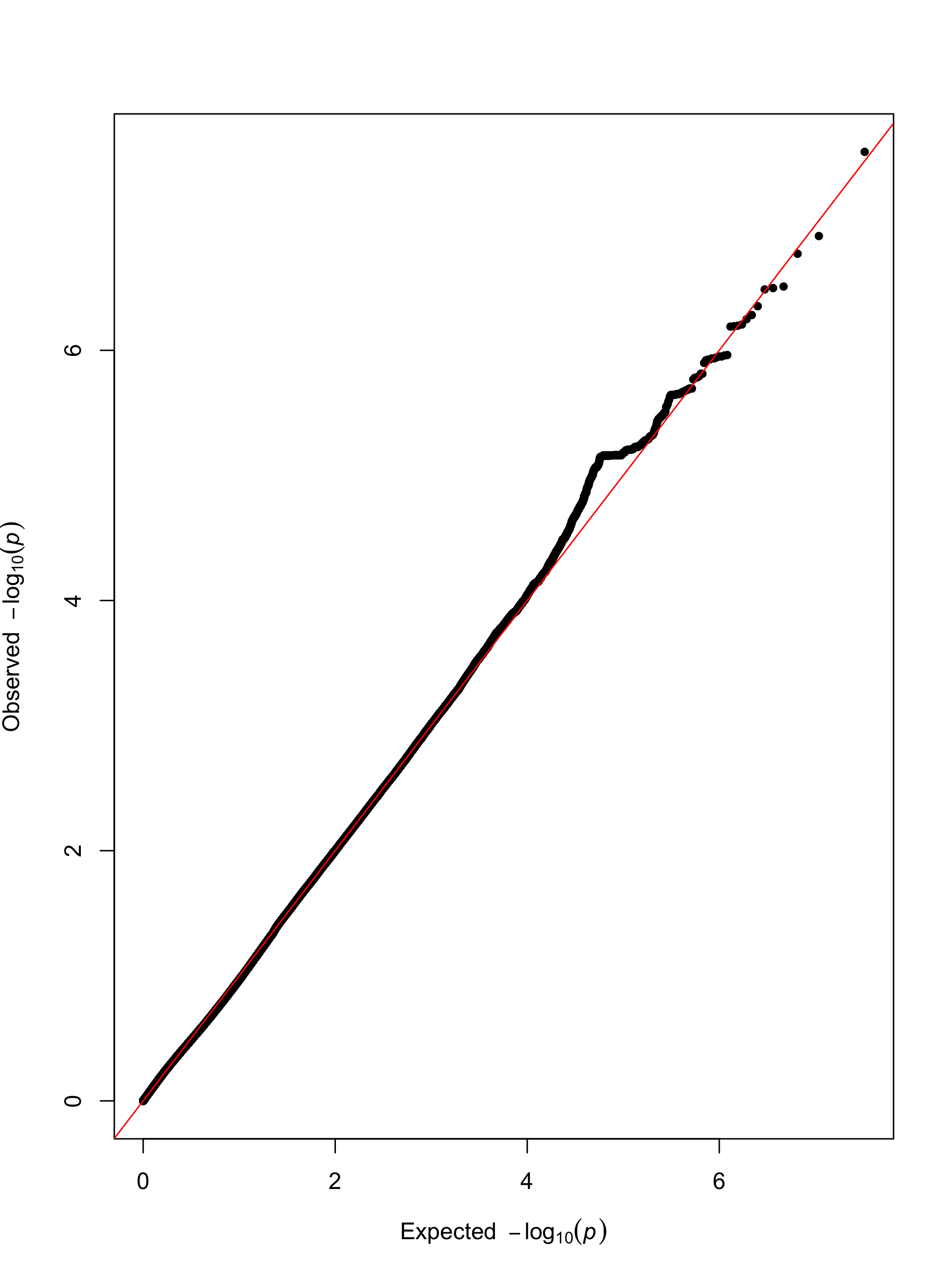

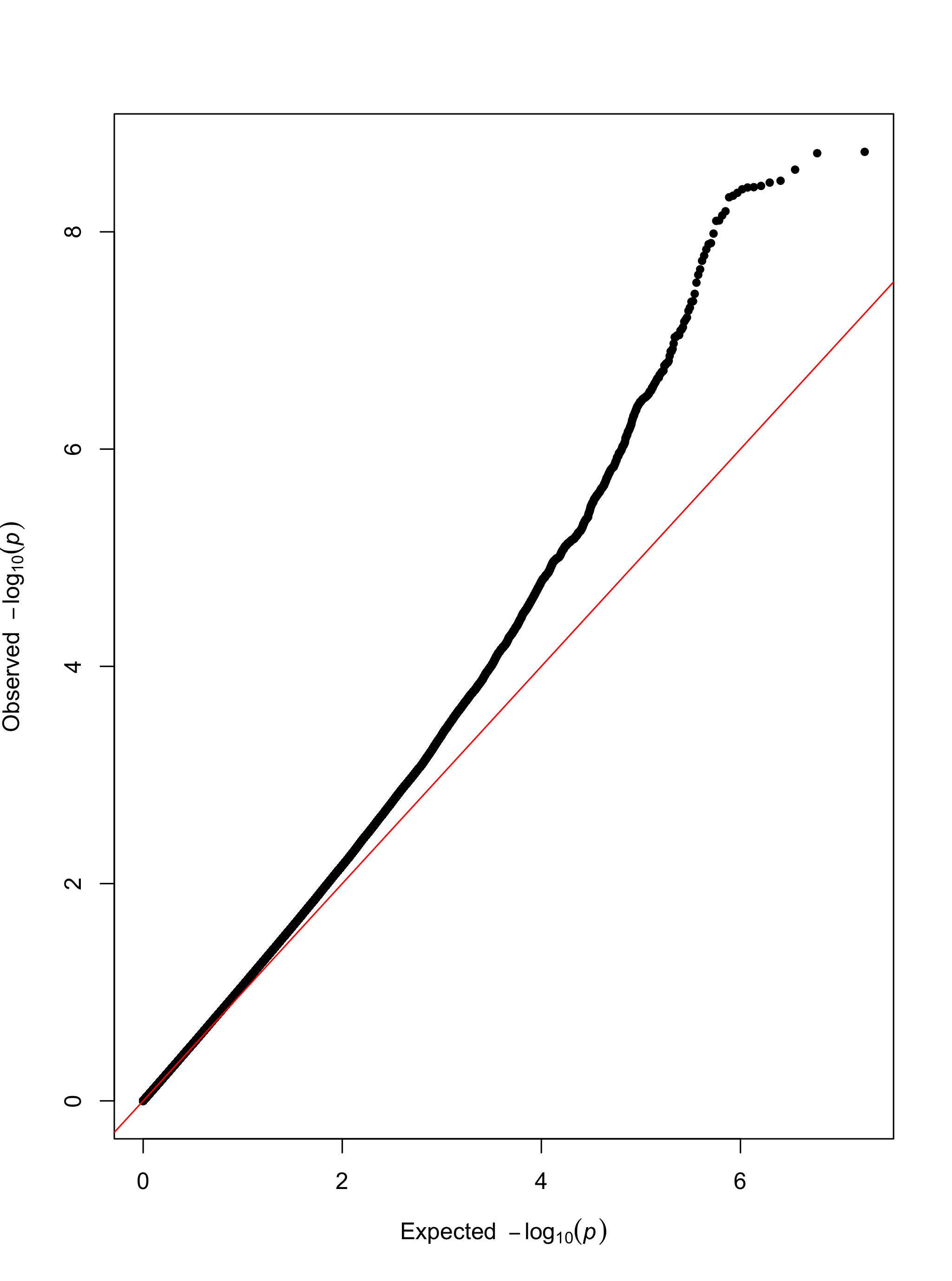


CUD

OUD


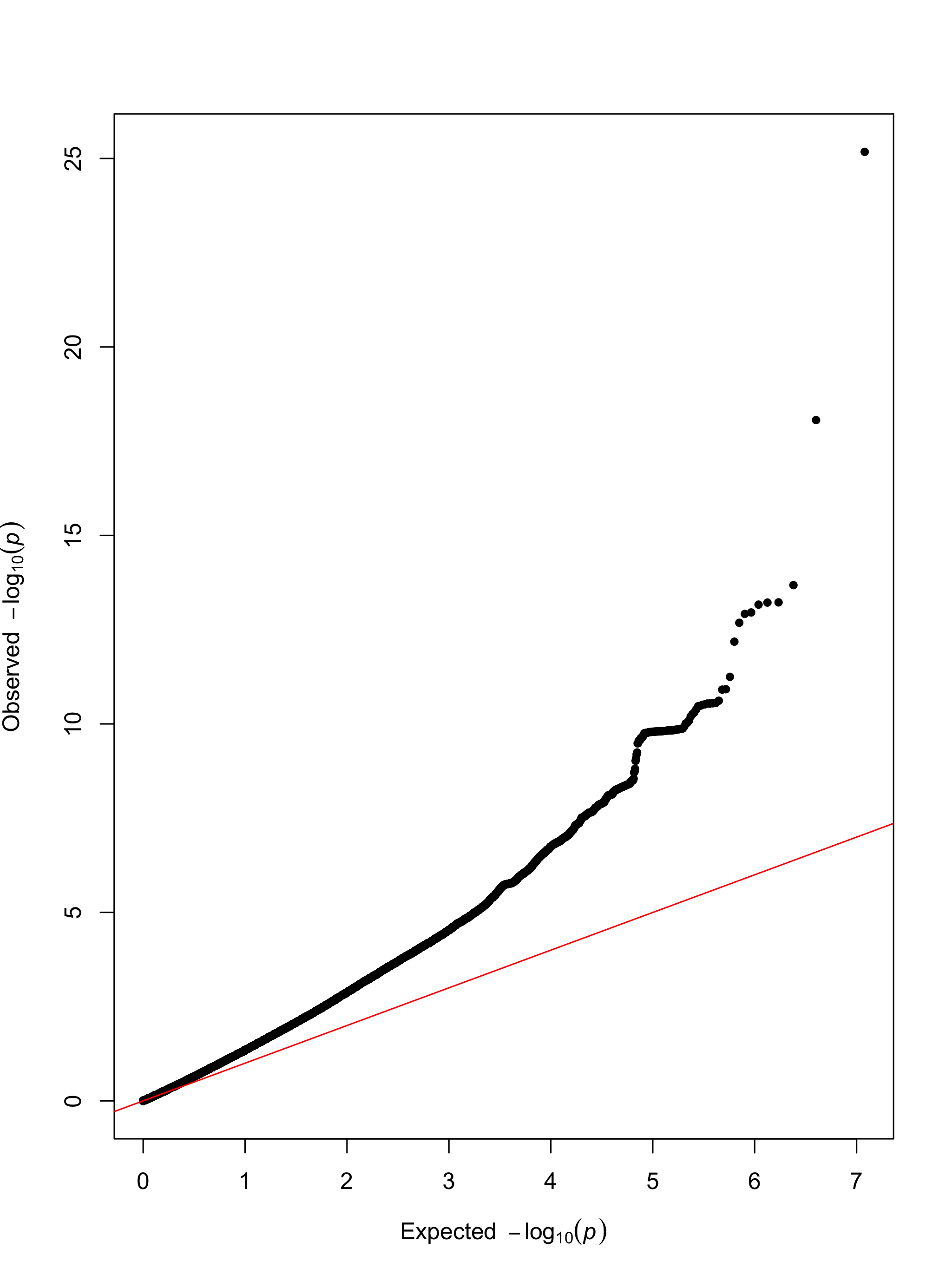


EXT

# Supplementary Figure S4. Q-Q plots of GWAS for attention deficit hyperactivity disorder (ADHD), conduct disorder (CD), antisocial personality disorder (ASPD), alcohol use disorder (AUD), opioid use disorder (OUD), cannabinoid use disorder (CUD) and the externalizing factor (EXT).

**References**

1. Warrier V, Kwong AS, Luo M, Dalvie S, Croft J, Sallis HM, et al. Gene-environment correlations and causal effects of childhood maltreatment on physical and mental health: a genetically informed approach. Lancet Psychiatry. 2021;**8**:373-86.

2. Demontis D, Walters RK, Martin J, Mattheisen M, Als TD, Agerbo E, et al. Discovery of the first genome-wide significant risk loci for attention deficit/hyperactivity disorder. Nat Genet. 2019;**51**:63-75.

3. Kurki MI, Karjalainen J, Palta P, Sipilä TP, Kristiansson K, Donner K, et al. FinnGen: Unique genetic insights from combining isolated population and national health register data. medRxiv. 2022.

4. Johnson EC, Demontis D, Thorgeirsson TE, Walters RK, Polimanti R, Hatoum AS, et al. A large-scale genome-wide association study meta-analysis of cannabis use disorder. Lancet Psychiatry. 2020;**7**:1032-45.
